# Geophagia among pregnant women: evaluating the microbiological and toxicological safety of calabash chalk and its implications on maternal health

**DOI:** 10.1007/s10653-025-02656-w

**Published:** 2025-07-30

**Authors:** Alice Njolke Mafe, Oluwadamilola Makinde, Rasheed Adegbola Adeleke

**Affiliations:** 1 Department of Biological Sciences, Faculty of Science, Taraba State University, Jalingo, Taraba State Nigeria; 2 Unit for Environmental Sciences and Management, North-West University (Potchefstroom Campus), Potchefstroom, South Africa; 3 Department of Biological Sciences and Biotechnology, Caleb University, Imota, Lagos State Nigeria

**Keywords:** Geophagia, Calabash chalk, Maternal health, Microbiological safety, Pesticides, Heavy metal contamination

## Abstract

Geophagia (i.e. calabash chalk consumption) is notably prevalent among pregnant women in parts of Africa and Asia. It is often used to alleviate pregnancy-related symptoms, such as nausea, a practice that carries complex cultural, nutritional, and health implications. This review examines the microbiological and chemical safety profiles of calabash chalk, emphasizing its potential impacts on maternal health. The cultural context of geophagia and perceived health benefits of calabash chalk consumption are explored, in addition to its mineral content, pesticide, heavy metal residues, and geographical variability in toxicity. Likewise, the balance between beneficial probiotics and pathogenic microorganisms, along with the potential risks these pose, to maternal health is assessed. This review also delves into the health risks associated with pesticide and heavy metal exposure, such as developmental toxicity and neurological impacts. Furthermore, potential nutritional benefits, including minerals such as calcium and iron, and its possible probiotic effects are discussed. Additionally, the review examines existing safety regulations, identifies gaps in monitoring and standards, and proposes directions for future research, particularly regarding the long-term effects of calabash chalk consumption during pregnancy. Overall, there is need for a balanced understanding of the risks and benefits of geophagia to promote maternal and foetal well-being.

## Introduction

Geophagia, the intentional consumption of soil, earth, or clay, is a long-standing practice with cultural, religious and medicinal significance across various societies (Abrahams & Parsons, 1997; Davies, 2023). It has been documented globally, particularly in Africa, Asia, and parts of the Americas, with evidence of its practice among ancient civilizations and indigenous tribes (Ford et al., 2020). Among its most frequent consumers are pregnant women, who may be drawn to this practice due to cultural beliefs and traditional recommendations (Aynalem et al., 2023). In Africa, geophagia is not only a culturally accepted behaviour but is also embedded within a framework of health beliefs that view soil consumption as beneficial during pregnancy (Madziva & Chinouya, 2023). For these communities, the practice of geophagia, particularly the consumption of types of clay soil such as calabash chalk (*Nzu*), is believed to address specific health needs associated with pregnancy (Bonglaisin et al., 2022; Public Health England, 2013). Other names by which edible clay is described include: *Mabele* in Congo, *Ebumba* in Uganda, *Shile* in Ghana and *Umcako* in South Africa (Abrahams et al., 2013). This makes geophagia an important area of study for insight of culturally rooted health practices and assessing the safety of such practices for maternal and foetal well-being (Cathrine Madziva et al., 2024).

**Fig. 1:**
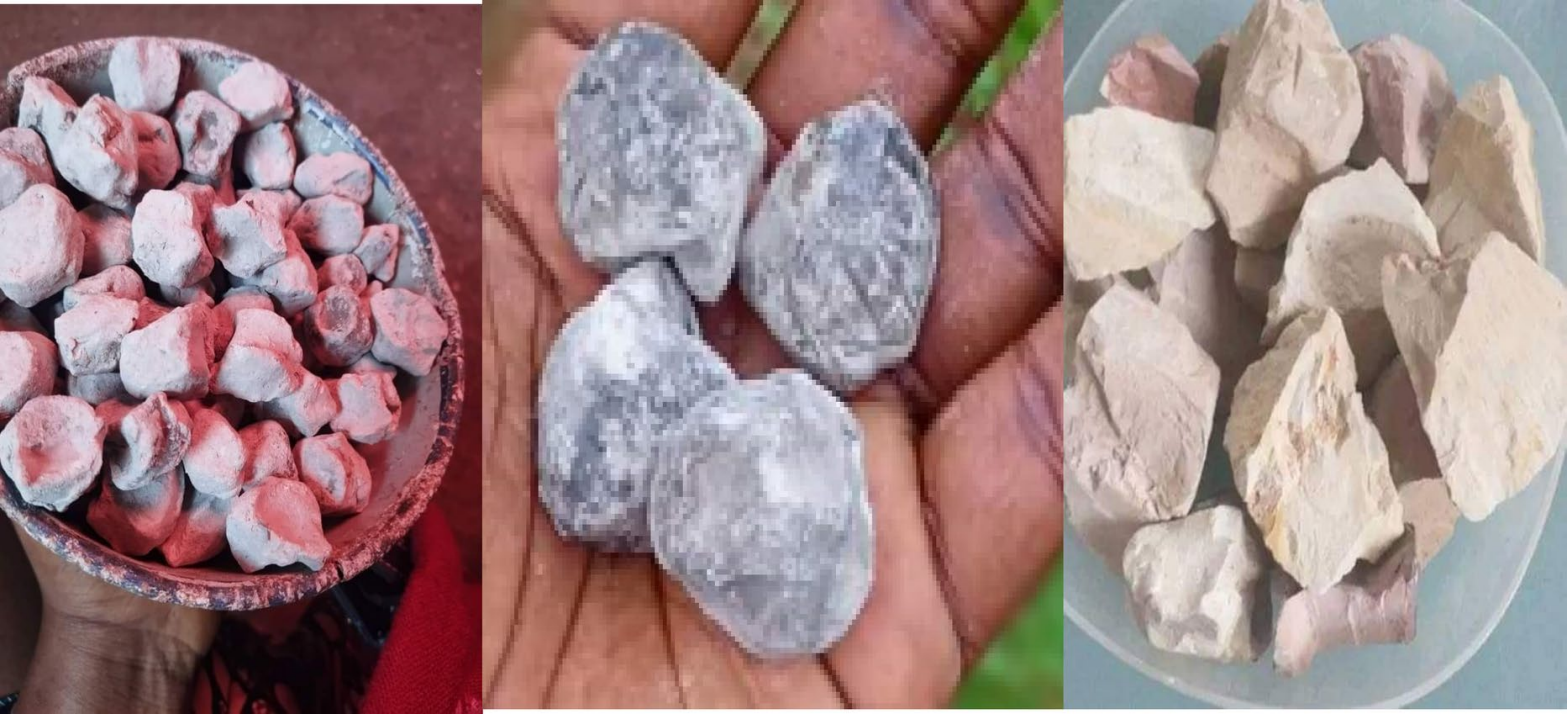
Calabash chalk

Calabash chalk is a kaolinite group mineral stone that occurs as fossilized seashell clay mined from shallow underground deposits. It can also be produced by moulding and firing mixtures of clay, mud, sand, wood and salt. Commercially, it is sold as powder, moulded pellets or blocks. Its principal constituent is aluminium silicate hydroxide (Al_2_Si_2_O_5_OH_4_), accompanied by trace elements including Ca, P, Mg, Zn, Cu, Mn and Fe (Ekong et al., 2024). Calabash chalk, a type of edible clay found in parts of Nigeria and other African regions (Fig. 1), is one of the preferred forms of soil consumed by pregnant women (Madziva & Chinouya, 2020). Traditional beliefs hold that calabash chalk consumption can relieve common pregnancy-related symptoms such as nausea, vomiting and heartburn (Mudonhi & Nunu, 2022). It is thought to calm the stomach, alleviate morning sickness and reduce acidity, providing comfort to expectant mothers (Lete & Alluέ, 2016). Also, some pregnant women consume calabash chalk for its mineral content, particularly as a source of calcium, magnesium, and iron, which are essential nutrients during pregnancy (Neelotpol et al., 2023). In communities with limited access to modern prenatal care, calabash chalk and similar clay-based substances are perceived as natural supplements that may help prevent mineral deficiencies (Oh et al., 2020). However, while the perceived benefits drive its use, there are also concerns regarding the potential health risks associated with its consumption, as clay may contain toxic substances and harmful microorganisms that could endanger both maternal and foetal health (Public Health England, 2013; Saha et al., 2021).

This review aims to explore microbiological and chemical contaminants of calabash chalk, particularly in the context of its consumption by pregnant women. The primary objective is to investigate the safety of calabash chalk by reviewing its microbiological and chemical profile, including pathogenic microorganisms, toxic heavy metals, and potentially harmful compounds that could lead to adverse health outcomes. This review will also assess existing literature to provide a detailed cognition of the health implications associated with calabash chalk consumption, examining both the potential benefits and the risks. By critically evaluating the microbiological and chemical safety of calabash chalk, this review seeks to inform healthcare providers, consumers and relevant government agencies on the potential health hazards of geophagia and encourage further research and policy directives into safer alternative supplements for pregnant women.

### The cultural context of geophagia

Geophagia, the practice of consuming soil or earth, is deeply rooted in cultural traditions and beliefs across various regions, particularly in parts of Africa, Asia, and Latin America. In many African societies like Nigeria, Ghana, and Cameroon, geophagia is linked to pregnancy, where women consume soils like calabash chalk to relieve nausea or address nutritional needs. (Frazzoli et al., 2016; Izugbara, 2004). In other cultures, geophagia may have ceremonial or spiritual significance, serving as a rite of passage, a fertility symbol, or a form of medicinal practice (Huebl et al., 2016). Table 1 emphasizes the need for further study on the safety and nutritional impact of soil consumption, especially in regions with a strong cultural association with this practice.

**Table 1:** The diversity of geophagic practices, emphasizing cultural significance, health impacts, and varying degrees of research findings across regions

Region	Type of soil consumed	Cultural significance	Health impacts	Findings	References
West Africa (Nigeria, Ghana)	*Nzu* (clay)	Common practice during pregnancy, believed to alleviate nausea and heartburn	Beneficial: Increases calcium intake, may relieve pregnancy symptoms	Some women experience anaemia or mineral imbalances due to contaminants	Babah et al. (2024)
East Africa (Kenya, Tanzania)	Clay, earth from riverbeds	Often consumed by pregnant women or those with mineral deficiencies	Harmful: Heavy metal exposure (lead, cadmium)	Limited studies on probiotic effects, but contamination with toxic metals found	Miller et al. (2018)
South Asia (India)	Clay or red soil	Often linked to traditional health beliefs, including a remedy for anaemia or pregnancy cravings	Potentially harmful due to exposure to bacteria and toxins	Soil from rural areas tested for microbial contamination	Lambraki et al. (2023)
South America (Brazil)	Red soil	Used for spiritual or medicinal purposes, particularly in Amazonian tribes	Mild: Some benefits reported in mineral absorption	Variability in soil composition, with some regions showing higher toxicity levels	Ellwanger and Chies, (2023)
Caribbean (Haiti, Jamaica)	Earth from termite mounds	Part of cultural rituals and believed to support digestion and relieve stomach problems	Beneficial: Supports digestion, but occasional gastrointestinal issues reported	Study showed both beneficial microbial flora and some harmful pathogens in soils	Auer et al. (2017)
Central Africa (Cameroon, Congo)	White clay	Used by pregnant women for nutrition and cravings	Beneficial: Provides trace minerals such as calcium, magnesium	Evidence of microbial pathogens present in some samples	Aprioku and Ogwo-Ude, (2018)
United States (Southern States)	Clay (especially from agricultural soils)	Used by some communities in rural areas for medicinal purposes or as a craving suppressant during pregnancy	Health risks: Heavy metals, e.g., arsenic and lead	Research shows variability in contamination across different regions	Alengebawy et al. (2021)
China (Guangxi Province)	Earth from riverbanks	Traditional use for digestion and balance of the body’s qi	Mild health benefits reported, but some risks from contamination	No significant health issues reported, but contamination concerns in urban soils	Alengebawy et al., (2021)
Mexico (Southern Mexico)	Red clay	Used for stomach ailments and general health maintenance	Beneficial in mild cases, but concerns about soil purity	Some beneficial minerals, but pesticides found in rural soils	Castrejón-Godínez et al. (2021)
Papua New Guinea	Earth from volcanic areas	Linked to traditional beliefs and considered to enhance strength and resilience	Mild health risks but also reported benefits in contending with calcium deficiencies	Inconsistent quality of soil, with some regions reporting higher mineral content, while others show contamination	Castrejón-Godínez et al. (2021)

## Chemical composition of calabash chalk

The chemical composition of calabash chalk varies widely depending on factors such as geographical location, environmental conditions, and methods of preparation (Aprioku & Ogwo-Ude, 2018). Various studies have documented a range of elements present in calabash chalk (see Table 2), including calcium, iron, magnesium, potassium, aluminum, silicon, and several trace elements. Analytical techniques like X-ray fluorescence (XRF), atomic absorption spectroscopy (AAS), and inductively coupled plasma mass spectrometry (ICP-MS) have been employed to characterize its composition. These studies reveal that the mineral and elemental content differs significantly across regions and samples, reflecting natural variability and potential external influences (Neelotpol et al., 2023). This section highlights the range of reported chemical constituents, providing a basis for later sections that examine their implications.

**Table 2:** Comparative analysis of mineral contents in calabash chalk vs. recommended daily intake for pregnant women

Mineral	Content in calabash chalk (mg per 100 g)	Recommended daily intake (Pregnant Women) (mg)	Relative contribution (%)	Comments	References
Calcium	10–100	1000–1300	Low	Limited bioavailability, may not meet daily needs	Oh et al. (2020)
Iron	5–50	27	Variable	Low absorption due to binding with soil compounds	Kocyłowski et al. (2018)
Magnesium	2–10	350–360	Minimal	Insufficient quantity to significantly contribute	Oh et al. (2020)
Potassium	1–5	2900–3000	Negligible	Present in trace amounts, unlikely to meet needs	Kocyłowski et al. (2018)

### Beneficial nutrients in calabash chalk

Geophagia, particularly the consumption of calabash chalk (a type of edible soil), is often culturally accepted and believed to provide certain nutritional benefits, especially for pregnant women (Bonglaisin et al., 2022; Davies, 2023). This section delves into the mineral composition of calabash chalk and evaluates whether its consumption can indeed fulfil some of the nutritional needs during pregnancy.

Studies on the mineral content of calabash chalk have identified several essential minerals, such as calcium, iron, magnesium, and potassium. These minerals are vital for bodily functions and may be absorbed via the gut (Bonglaisin et al., 2022; Ekong et al., 2015), providing some benefits for pregnant women. Calcium is important to foetal bone development, and largely deficient in pregnant women’s diets. Calabash chalk is believed to be a good source of calcium, helping to support bone health (Kumar & Kaur, 2017). Iron is critical to preventing anaemia, especially during pregnancy, iron is another mineral reportedly found in calabash chalk. Pregnant women require additional iron to support increased blood volume and foetal development (Georgieff, 2020). Magnesium mineral plays a role in numerous enzymatic reactions and muscle function, including uterine relaxation during pregnancy (Marín et al., 2023). Potassium is essential for maintaining fluid and electrolyte balance, potassium may support maternal health by regulating blood pressure in pregnant women (Iqbal et al., 2019).

### Non-beneficial chemicals in calabash chalk

#### Heavy metals

While calabash chalk is traditionally consumed for its purported health benefits, studies have shown that it contains harmful heavy metals and toxins that pose significant health risks, especially to pregnant women and their developing foetuses (Table 3).

**Table 3:** Comparison of heavy metal levels in calabash chalk from selected regions, sources of contamination and health risk

Region	Type of calabash chalk	Lead (Pb) (mg/kg)	Cadmium (Cd) (mg/kg)	Arsenic (As) (mg/kg)	Mercury (Hg) (mg/kg)	WHO Standard (mg/kg)	Source of contamination	Health risks identified	References
Nigeria (South East)	Clay (*Nzu*) from local areas	0.2–3.5	0.05–1.2	0.1–1.4	0.01–0.1	Pb: 0.01, Cd: 0.01, As: 0.01, Hg: 0.001	Agricultural runoff, local industrial activities	Developmental issues, neurological effects, cancer risks from long-term exposure	Orisakwe et al. (2020)
Ghana (Ashanti Region)	Clay from riverbanks	0.1–1.8	0.04–0.8	0.08–1.2	0.03–0.09	Pb: 0.01, Cd: 0.01, As: 0.01, Hg: 0.001	Agricultural and mining activities	Neurological issues, kidney damage, foetal developmental risks	Orisakwe et al. (2020)
Uganda (Central Region)	Clay from rural soils	0.5–4.0	0.03–0.5	0.1–2.3	0.02–0.1	Pb: 0.01, Cd: 0.01, As: 0.01, Hg: 0.001	Natural soil composition, agricultural fertilizers	Growth retardation, cognitive impairment, cancer potential	Johan et al. (2021)
Tanzania (Zanzibar)	White clay	0.05–1.5	0.03–0.6	0.05–1.0	0.01–0.05	Pb: 0.01, Cd: 0.01, As: 0.01, Hg: 0.001	Local agricultural use	Reproductive toxicity, liver damage	Johan et al. (2021)
South Africa (Eastern Cape)	Red clay	0.1–2.5	0.02–0.6	0.1–1.5	0.02–0.07	Pb: 0.01, Cd: 0.01, As: 0.01, Hg: 0.001	Agricultural and urban runoff	Heavy metal toxicity, gastrointestinal problems	Agoro et al. (2020)
Kenya (Coastal Region)	Clay from coastal soils	0.3–2.0	0.04–0.8	0.2–1.0	0.02–0.1	Pb: 0.01, Cd: 0.01, As: 0.01, Hg: 0.001	Use of pesticide-laden agricultural soils	Developmental delays, immune system suppression	Agoro et al. (2020)
Cameroon (Centre Region)	White clay	0.1–1.2	0.02–0.4	0.07–1.0	0.01–0.05	Pb: 0.01, Cd: 0.01, As: 0.01, Hg: 0.001	Mining and agricultural runoff	Kidney damage, cognitive impairment in children	Aprioku and Ogwo-Ude, (2018)
India (Rural Areas)	Red clay from riverbeds	0.05–2.3	0.01–0.5	0.04–0.8	0.02–0.06	Pb: 0.01, Cd: 0.01, As: 0.01, Hg: 0.001	Local agricultural practices	Gastrointestinal problems, high risk for pregnant women	Aprioku and Ogwo-Ude, (2018)
Haiti (Caribbean)	Clay from termite mounds	0.1–1.5	0.02–0.4	0.1–1.0	0.01–0.05	Pb: 0.01, Cd: 0.01, As: 0.01, Hg: 0.001	Local soil from rural farming areas	Liver damage, developmental delays, increased risk of miscarriage	Chisanga et al. (2020)
Brazil (Amazon Region)	Clay from forested areas	0.3–2.2	0.04–0.6	0.1–1.4	0.03–0.1	Pb: 0.01, Cd: 0.01, As: 0.01, Hg: 0.001	Mining activities, organic pollution	Risk of organ toxicity, increased blood pressure	Acioly et al. (2024)
Indonesia (Asia)	Java soil	–	–	1.8–9.2	–	Pb: 0.01, Cd: 0.01, As: 0.01, Hg: 0.001	Natural soil composition	Developmental issues, neurological effects, cancer risks from long-term exposure	Mahaney et al. (2000)

Heavy metals such as lead, mercury, cadmium, and arsenic are frequently identified in calabash chalk samples. These metals are naturally present in the earth's crust but become toxic when they accumulate in high concentrations in the human body (Singh et al., 2022). Lead exposure is highly toxic, particularly to developing foetuses. It can cross the placenta and accumulate in foetal tissues, causing neurodevelopmental issues, lower IQ, and learning disabilities. For pregnant women, lead exposure can lead to anaemia, hypertension, and kidney dysfunction (Gundacker et al., 2021). Mercury is another neurotoxic element that poses severe health risks, especially to foetuses. Prenatal mercury exposure can impair brain development, motor skills, and cognitive abilities. Chronic exposure in adults can lead to tremors, memory loss, and emotional instability (Dack et al., 2022). Cadmium exposure has been linked to kidney damage, bone demineralization, and cardiovascular issues. For pregnant women, cadmium exposure may also result in low birth weight and preterm birth. Cadmium's long half-life in the body means it can accumulate over time, increasing toxicity risks (Genchi et al., 2020a, b). Arsenic is known to be carcinogenic, hence arsenic exposure can cause skin lesions, cancer, and cardiovascular diseases. For pregnant women, even low-level arsenic exposure can increase the risk of foetal growth restrictions, adverse birth outcomes, and developmental problems (Ortiz-Garcia et al., 2023).

#### Pesticide residues

Calabash chalk, in similarity to many types of soil, can be exposed to environmental contaminants, including pesticide residues, through agricultural runoff, industrial activities, and atmospheric deposition (Mishra et al., 2023). These contaminants pose additional health risks for those who consume calabash chalk, especially pregnant women, who are more susceptible to chemical exposure risks for themselves and their developing foetuses (Ahmad et al., 2024).

While environmental contamination from nearby agricultural activities is a plausible source of pesticide residues in calabash chalk, current scientific literature does not provide direct evidence confirming the presence of modern pesticide residues in analyzed calabash chalk samples. Theoretical pathways, such as pesticide runoff, aerial deposition, or improper waste disposal, remain valid concerns. However, chemical analyses performed on calabash chalk have primarily detected heavy metals rather than conventional pesticides. For example, a study that examined the chemical composition of calabash chalk and its effects on locomotor activity in mice reported the presence of toxic elements like lead (19.26 ppm), chromium (34.73 ppm), and arsenic (4.57 ppm), but no pesticide residues were identified (Neelotpol et al., 2023). Another study focusing on gestational toxicity in Wistar rats assessed maternal and fetal outcomes following calabash chalk exposure but did not include or report pesticide detection as part of its analysis (Aprioku & Ogwo-Ude, 2018). Furthermore, a more comprehensive chemical analysis utilizing pressurized fluid extraction followed by gas chromatography-mass selective detection (GC-MSD) identified persistent organic pollutants (POPs) in calabash chalk, including alpha-lindane, endrin, endosulfan II and p,p′-DDD compounds historically used as pesticides but now classified as long-banned, highly persistent pollutants (Dean et al., 2004). These findings suggest that while legacy contaminants may be present, there is currently no documented detection of commonly used modern pesticides in calabash chalk.

The following are examples of pesticides commonly found as soil residues and known to pose health risks (Table 4):

**Table 4:** Common pesticide residues in calabash chalk and associated health risks

Pesticide class	Examples	Potential health effects	References
Organochlorines	DDT, aldrin	Endocrine disruption, carcinogenicity, neurotoxicity	Algharably et al. (2023)
Organophosphates	Chlorpyrifos	Neurotoxicity, developmental delays	Algharably et al. (2023)
Carbamates	Aldicarb	Nervous system effects, hormonal disruptions	Nasrabadi et al. (2024)
Pyrethroids	Permethrin	Respiratory issues, potential immune suppression	Chrustek et al. (2018)

Organochlorine Pesticides (OCPs): These pesticides, such as DDT and aldrin, are persistent organic pollutants that resist environmental degradation. Organochlorines can bioaccumulate in the human body, leading to long-term exposure risks, even from small doses (Ansari et al., 2024).

Organophosphates: Used in agriculture for pest control, organophosphate pesticides such as chlorpyrifos are neurotoxic and can impair nervous system function. These chemicals pose particular risks to foetuses, leading to potential developmental issues and cognitive impairments (Neylon et al., 2022).

Carbamates and Pyrethroids: While generally less toxic than organophosphates, carbamates and pyrethroids can still affect the nervous system and have been linked to respiratory issues and hormonal disruptions, especially in prolonged exposures (Kaur et al., 2024).

## Experimental toxicological studies on calabash chalk

A recent cross-sectional study also reported a high prevalence of kaolin consumption among migrant women living in urban France (Caillet et al., 2019), highlighting its global relevance. Despite the widespread practice of calabash chalk consumption, especially among pregnant women, there remains a significant gap in the availability of quantitative toxicological data necessary to establish exposure thresholds and health risks. Recent in vivo and in vitro laboratory studies have attempted to bridge this gap by investigating the acute and chronic toxic effects of calabash chalk, focusing on endpoints such as LD_50_ (lethal dose), NOAEL (No Observed Adverse Effect Level), LOAEL (Lowest Observed Adverse Effect Level), and target organs affected.

(Alelign et al., 2020) conducted an acute toxicity study using albino rats administered with calabash chalk commonly consumed in Douala, Cameroon. The results indicated a relatively high oral LD₅₀ value of 5600 mg/kg, suggesting low acute toxicity under single-dose exposure conditions. However, signs of hepatotoxicity and renal stress were observed at repeated sub-lethal doses. Histopathological examination revealed hepatic congestion, glomerular atrophy, and mild intestinal mucosal damage.

Similarly, a recent study by Nnamdi Kingsley Okore et al., (2024) reported dose-dependent alterations in hematological and biochemical markers in rats administered with locally sourced calabash chalk for 28 days. The NOAEL was established at 100 mg/kg body weight/day, while the LOAEL was observed at 250 mg/kg, where significant alterations in liver enzymes, serum creatinine, and reproductive hormones were recorded. The most affected organs included the liver, kidneys, and reproductive system—a particularly critical concern in the context of maternal health.

These findings highlight the need for further comprehensive studies to validate toxic thresholds across different populations and to account for factors such as bioavailability, elemental composition, and co-exposure with other contaminants. Additionally, the lack of data on chronic low-dose exposure—the most likely scenario in geophagic users—remains a major limitation in current toxicological risk assessments. A summary of experimental toxicological studies on calabash chalk, including key metrics such as LD₅₀, NOAEL, LOAEL, and affected target organs, is presented in Table 5.

**Table 5:** Summary of experimental toxicological studies on calabash chalk

Model/species	Dose/duration	LD₅₀ (mg/kg)	NOAEL (mg/kg)	LOAEL (mg/kg/day)	Observed effects	Target organs	References
Wistar rats (acute oral)	Single dose	5600	Not reported	≥ 1,000	Mild hepatotoxicity, renal stress, intestinal mucosal changes	Liver, kidneys, intestines	Chinko et al. (2022)
Albino rats (subchronic)	28 days oral	Not reported	100 mg/kg/day	250	Elevated liver enzymes, reduced sperm count, hormonal imbalance	Liver, kidneys, reproductive system	Kale et al. (2019)
Mice (oral exposure)	14 days	Not reported	50 mg/kg/day	150	Disrupted haematological profile, reduced feed intake	Blood, GI tract	Ismail et al. (2023)

## Microbiological profile of calabash chalk

Calabash chalk and its microbiological profile has been studied to understand its potential health implications. The microbiological profile of calabash chalk encompasses a variety of microorganisms, including both beneficial and potentially harmful species. Recognizing the diversity and balance of these microbial populations is crucial, especially considering calabash chalk 's consumption by pregnant women, who may be more vulnerable to infections as their immune system is suppressed (Aprioku & Ogwo-Ude, 2018).

### Beneficial microorganisms

Some geophagic soils, including calabash chalk, may contain microorganisms that offer potential health benefits, similar to those found in probiotics Beneficial microbes can support digestive health, improve gut microbiota balance, and potentially provide immune support (Cuesta et al., 2021). While research on specific probiotic strains in calabash chalk is limited, studies on other geophagic clays and natural soils have found microbial populations such as *Lactobacillus sp.* and *Bifidobacterium sp.* which are known for their positive impacts on digestive health (Abdelhamid et al., 2019). Potential probiotic benefits that could accrue to consumers of calabash chalk if it contains beneficial bacteria include, maintenance or restoration gut flora balance, which is particularly beneficial to pregnant women dealing with digestive issues such as nausea or constipation (Obuchowska et al., 2022). Also, competitive exclusion of pathogens by certain beneficial bacteria can prevent the growth of pathogens. This occurs by outcompeting pathogens for nutrients and space or by producing antimicrobial substances, potentially reducing the risk of infections from soil-borne pathogens (Bonaterra et al., 2022).

### Pathogenic microorganisms

In addition to potentially beneficial microbes, calabash chalk can also harbour harmful pathogens. Pathogens commonly found in soil include bacteria, fungi, and parasites, which can pose significant health risks to consumers. Pregnant women, whose immune systems may be suppressed, are particularly susceptible to these risks, which can include foodborne illnesses and complications affecting both mother and foetus (Mate et al., 2021).

#### Common soil-borne pathogens

Pathogenic bacteria such as *Escherichia coli*, *Salmonella sp.* and *Clostridium* species are known soil contaminants (Nieder et al., 2018). These bacteria can cause severe gastrointestinal infections, sometimes leading to complications in pregnancy such as dehydration, malnutrition, or preterm labour. Also, soil may also contain fungal spores, such as those from *Aspergillus* species, which can produce mycotoxins harmful to human health. Mycotoxin exposure can lead to liver and kidney damage and, in severe cases, may impact foetal development (Gwinn et al., 2023). In addition, soil-transmitted helminths such as *Ascaris sp.* and *Trichuris sp.* are also common in certain soils. Ingesting these parasites can result in intestinal infections, malnutrition, and anaemia, which may have further complications during pregnancy (Caldrer et al., 2022). Table 6 presents confirmed soil-borne pathogenic microorganisms identified in various natural clays and calabash chalk, along with their associated health risks upon consumption. Consumption of calabash chalk without proper treatment (e.g., sterilization or pasteurization) could expose individuals to these pathogens. Soil-borne diseases are particularly concerning in pregnant women, as infections could result in adverse pregnancy outcomes, including miscarriage, stillbirth, or congenital infections.

**Table 6:** Pathogenic microorganisms identified in natural clays and calabash chalk with associated health risks

Type of clay or calabash chalk	Pathogenic microorganisms identified	Associated health risks	Notes on microbial activity	References
Kisameet Clay (British Columbia, Canada)	*Candida albicans, Cryptococcus neoformans, Mycobacterium marinum* (tested organisms)	Not ingested; evaluated for treatment of MDR infections	Exhibits strong antimicrobial and antibiofilm activity	Behroozian et al. (2020)
White clay samples (used in geophagia)	*Staphylococcus aureus, Bacillus cereus*	GI upset, opportunistic infections, skin infections	Survive dry clay matrices and resist gastric conditions	Azmi et al. (2021)
Bentonite clay (commercial detox product)	*Pseudomonas aeruginosa, E. coli, Candida tropicalis*	Gut and systemic infections in immunocompromised individuals	Cultivable pathogens found in marketed samples	Moosavi (2017)
Termite mound clay (used in traditional medicine)	*Salmonella typhi, Streptococcus sp., Candida albicans*	Typhoid fever, gut inflammation, candidiasis	Microbial risks from soil and faecal matter	Mahamat et al. (2021)
Bauxite-rich clay (West Africa)	*Bacillus subtilis (toxigenic), Clostridium perfringens, E. coli*	GI disturbances, food poisoning, toxin release	Traditional roasting ineffective at full sterilization	Grenda et al. (2023)
Nzu (Nigeria, West Africa)	*Fusarium sp.*	Mycotoxicosis (liver/kidney damage), foetal developmental issues	Ingestion of contaminated soil	Aleruchi et al. (2022)
Geophagic clay (Southern and Western Nigeria)	*Ascaris sp., Trichuris sp.*	Malnutrition, anaemia, low birth weight, preterm birth	Ingestion of contaminated soil	Bisi-Johnson et al. (2013)

Beneficial bacteria, such as *Lactobacillus sp.*, are highlighted for their role in improving gut health and preventing infections (Zhang et al., 2015). However, the presence of harmful bacteria, such as *Escherichia coli*, raises concerns due to their potential to cause gastrointestinal infections. Besides, fungi such as *Aspergillus sp.* are noted for producing mycotoxins, which can result in severe liver or kidney damage (Kotun et al., 2024; Mafe & Büsselberg, 2024a, 2024b; Makinde et al., 2020). Table 6 also lists parasites such as *Ascaris lumbricoides*, which may contribute to malnutrition, anaemia, and intestinal blockages. This profile underscores the dual nature of microbial presence in calabash chalk, emphasizing the need for careful evaluation to mitigate health risks while leveraging potential benefits.

## Health implications and risk factors in calabash chalk consumption by pregnant women/foetus

Calabash chalk, a type of edible clay, is consumed in many cultures for its perceived health benefits, particularly by pregnant women. However, alongside its traditional appeal, concerns have emerged regarding the potential health risks associated with its consumption. Calabash chalk may contain harmful contaminants, such as heavy metals, toxins, and pathogens, which could pose significant risks to both maternal and foetal health. This section highlights the associated risks, including potential toxicity and nutrient absorption interference, emphasizing the need for awareness and informed decision-making for maternal well-being (ter Borg et al., 2023).

Pesticide residues in soil consumed as calabash chalk pose both acute and chronic health risks. Pregnant women are particularly vulnerable, as chemical exposures can affect both maternal health and foetal development (Ventura-Miranda et al., 2022). In general, most pesticides act as endocrine disruptors, interfering with hormone systems that regulate foetal development. Such interference may lead to birth defects, low birth weight, and developmental delays (Puche-Juarez et al., 2023). Neurotoxic pesticides, particularly organophosphates and carbamates, can disrupt foetal brain development, potentially resulting in cognitive impairments, motor skill delays, and behavioural changes in exposed children (Richardson et al., 2019). Certain pesticides, such as some organochlorines, are classified as probable or possible human carcinogens. Long-term exposure increases the risk of cancer, affecting organs such as the liver, lungs, and kidneys (Cavalier et al., 2023). Prolonged pesticide exposure can weaken the immune system, making both mothers and foetuses more susceptible to infections and diseases (Prahl et al., 2021).

Furthermore, the consumption of calabash chalk (edible clay) during pregnancy has also been linked to various potential health risks, particularly due to the presence of heavy metals (Table 7). Heavy metals such as lead, mercury, cadmium, and arsenic, commonly found in soils, can have detrimental effects on both maternal and foetal health (Vofo et al., 2019).

**Table 7:** Potential health impacts of metals resulting from calabash chalk consumption

Heavy metal	Maternal health	Foetal health	Mechanisms of action	References
Lead (Pb)	Lead exposure during pregnancy increases the risk of high blood pressure, gestational hypertension, preeclampsia, and can interfere with calcium metabolism, potentially leading to weakened bones and other disruptions	Lead is highly toxic to developing foetuses. It impairs brain development, leading to cognitive deficits, learning disabilities, and behavioural problems. It also increases the risk of premature birth, low birth weight, and stillbirth	Lead disrupts neurotransmitter function, impairs DNA synthesis, and interferes with enzymatic activities crucial for foetal growth and development	Zhong et al. (2022)
Mercury (Hg)	Mercury exposure during pregnancy can cause neurological and kidney damage, leading to tremors, mood disorders, and memory loss	Mercury is harmful to the nervous system, causing developmental delays, sensory impairments, and motor dysfunctions. It can lead to cerebral palsy or foetal death and can cross the placenta to accumulate in the brain	Mercury inhibits protein synthesis, causes oxidative stress, and damages cells and tissues, especially in the foetal brain, where neurogenesis and synaptogenesis occur	Dack et al. (2022)
Cadmium (Cd)	Cadmium exposure is associated with kidney damage, bone demineralization, and increased risk of hypertension. It can accumulate in the liver and kidneys, causing long-term health issues	Cadmium is a teratogen, causing birth defects, intrauterine growth restriction (IUGR), low birth weight, and abnormalities in the development of the skeleton, liver, and brain. It crosses the placenta and disrupts oxygen and nutrient supply to the foetus	Cadmium induces oxidative stress, interacts with metal ions, and disrupts cellular processes, affecting enzymes involved in cell growth and repair	Satarug (2024)
Arsenic (As)	Chronic arsenic exposure can cause skin lesions, liver damage, cardiovascular disease, and increase cancer risk. Its toxicity may be amplified during pregnancy due to altered metabolism	Arsenic can cross the placenta, resulting in developmental delays, birth defects, cognitive deficits, immune dysfunction, and abnormal lung development. It increases the risk of childhood cancers	Arsenic induces cellular apoptosis (programmed cell death), oxidative damage, and interferes with DNA repair mechanisms, leading to mutations and developmental abnormalities	Genchi et al., (2020a, 2020b)

Pregnant women are particularly vulnerable to infections due to their altered immune systems (Kumar et al., 2022). This makes them more susceptible to the negative health impacts of pathogenic microorganisms from geophagic practices (Table 8). The implications for maternal and foetal health can be severe but not limited to gastrointestinal infections as a consequence of ingesting pathogenic bacteria such as *E. coli* and *Salmonella sp.* contaminating calabash chalk. This can result in dehydration and electrolyte imbalances, which increase the risk of complications such as preterm labour or foetal distress. Systemic infections caused by pathogenic bacteria such as *Listeria sp.* or *Salmonella sp.*, enter the bloodstream (bacteraemia), cross the placental barrier and cause foetal infections, increasing the risk of miscarriage, stillbirth, or premature birth (Chan & Smith, 2018). Mycotoxins (secondary metabolites (Mafe & Büsselberg, 2024a, b) produced by fungi in calabash chalk, such as Aflatoxin and Ochratoxin, can cause liver damage, immune suppression, and developmental toxicity. In pregnant women, mycotoxin exposure has been linked to increased risks of preterm labour, low birth weight, and foetal developmental defects (Kyei et al., 2020). Parasitic infections caused by soil-transmitted helminths can cause chronic malnutrition, anaemia, and intestinal blockages. For pregnant women, these infections can impair nutrient absorption, leading to deficiencies that negatively impact both maternal health and foetal development. Severe cases of parasitic infections can lead to preterm birth or stillbirth (Davies, 2023).

### Strategies to mitigate health risks

To minimize the health risks associated with calabash chalk consumption, several strategies can be adopted. Sterilization of calabash chalk is one effective method to reduce pathogen transmission is through sterilization or pasteurization of calabash chalk before consumption. This could be done by heating the soil to high temperatures or through UV treatment to kill harmful microorganisms (Yemmireddy et al., 2022). In addition, vendors especially those in the rural areas should be educated on proper handling and hygienic practices. This includes proper handwashing and clean storage of calabash chalk, to help prevent pathogen transmission (Tenebe et al., 2023). Also, the soil source of calabash chalk should be frequently tested to ensure low contamination levels associated with heavy metals and pesticides that could lead to long term risks to consumers. A good monitoring and regulatory framework for geophagic practices, including quality standards for soil consumption, could help ensure that calabash chalk is safe for human consumption. A summary of studies documenting infections and health complications associated with soil-borne pathogenic microorganisms, with a focus on pregnancy outcomes, is presented in Table 8.

**Table 8:** Summary of studies documenting infections and health complications associated with pathogenic microorganisms found in soil, focusing on pregnancy outcomes

Country	Pathogenic microorganisms identified	Infections/Health complications	Outcomes specific to pregnancy	References
Nigeria	*Clostridium perfringens, Salmonella sp.*	Gastrointestinal infections, sepsis	Preterm birth, low birth weight, miscarriage	Rosso et al. (2023)
Kenya	*Escherichia coli, Listeria monocytogenes*	Urinary tract infections, foetal meningitis	Increased risk of preterm birth, stillbirth, foetal growth restriction	Bæk et al. (2019)
India	*Aspergillus sp., Candida albicans*	Fungal infections, systemic mycosis	Maternal respiratory complications, increased risk of congenital infections	Grassi et al. (2024)
Brazil	*Toxoplasma gondii*	Toxoplasmosis infection	Miscarriage, congenital anomalies, stillbirth	Alvarado-Esquivel et al. (2015)
Mexico	*Enterococcus sp., Staphylococcus aureus*	Bacterial vaginosis, urinary tract infections	Increased risk of preterm labour, low birth weight, foetal infection	Daskalakis et al. (2023)
USA	*Campylobacter jejuni, Vibrio cholera*	Gastrointestinal infection, cholera	Increased risk of dehydration, preterm labour, foetal distress	El Hayek et al. (2023)
Thailand	*Trichomonas vaginalis, Giardia lamblia*	Parasitic infections, intestinal inflammation	Preterm birth, low birth weight, neonatal infection	Wongstitwilairoong et al. (2023)

## Gaps in research

### Chemical profiles in calabash chalk

Despite known risks associated with chemicals such as pesticide and heavy metal contaminants, there are several gaps in research on pesticide residues in calabash chalk and related health implications for consumers (Ngole-Jeme et al., 2018; Wyckhuys et al., 2020). These gaps could be addressed with focus on determining region- specific pesticide and heavy metal profiles in calabash chalk. This would provide insight into environmental contamination risks based on geographical factors and agricultural practices (Parven et al., 2024). Soil composition can vary widely based on geographic location, underlying geology, and environmental exposure, such as proximity to agriculture or industrial areas. Mapping pesticide and heavy metal profiles specific to different regions within a country, or across different countries, would provide more accurate risk assessments for consumers in each area. Such research could help establish region-based safety guidelines or even promote safer sources of calabash chalk based on lower contaminant levels (Guimarães et al., 2024).

Also, while major heavy metals such as lead, cadmium, and mercury are often studied, there is minimal information on rare trace elements that may be present in calabash chalk. Elements such as thallium, vanadium, and antimony, though found in lower concentrations, can still be toxic, especially with chronic exposure (Ekong et al., 2015). Examining the presence and health implications of these less-studied elements would contribute to a more comprehensive safety profile of calabash chalk and highlight potential risks that might be overlooked in routine screenings (Gasull et al., 2024).

In addition, safe thresholds for heavy metals and pesticides specific to geophagic soils are non-existent. Most existing safety thresholds for heavy metals and pesticides are developed based on food and water, not soils that are deliberately ingested. Establishing safe consumption limits specific to geophagic soils would provide clearer guidelines for individuals who practice geophagia, particularly pregnant women who may have different susceptibility to contaminants. Such thresholds would consider both chronic low-dose exposure and potential cumulative effects (Adil et al., 2023).

Similarly, developing a standardized testing protocol specifically for calabash chalk would address the unique risks of this geophagic practice. A testing framework could include comprehensive screening for heavy metals, pesticides, trace elements, and microbial contaminants, with an emphasis on factors such as regional origin and environmental influences (Oyebanjo et al., 2020). This framework could serve as a model for assessing other types of geophagic soils worldwide, improving safety protocols for a traditionally underregulated practice.

**Fig. 2:**
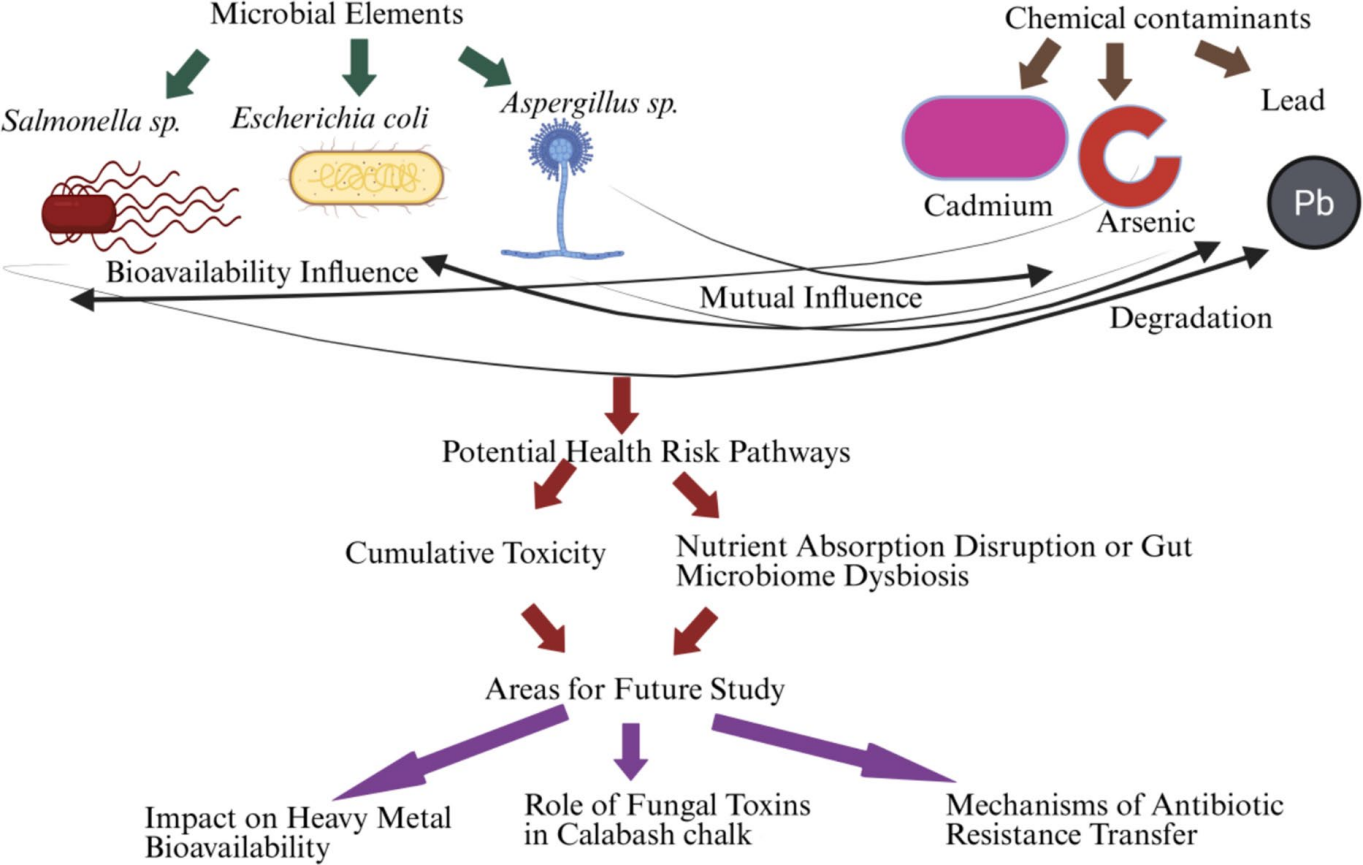
A hypothetical model showing interactions between microbial and chemical elements in calabash chalk

Furthermore, the interactive effects of pesticides and heavy metals and microbial contaminants present in calabash chalk are not well understood. Recognizing how chemical and microbiological components may interact to increase or mitigate health risks is essential for a holistic safety assessment (Wallace & Buha Djordjevic, 2020). For instance, certain metals may affect microbial activity or enhance pathogen survival, while microbial interactions with soil minerals could influence metal bioavailability (Ahmed et al., 2019). This area of study could lead to insights on potential synergistic or antagonistic effects between chemical and biological contaminants, which is critical for accurate risk evaluation. Figure 2 presents a hypothetical model showing interactions between microbial and chemical elements in calabash chalk illustrating a network of interactions among critical components of calabash chalk, accentuating how microbial elements such as bacteria (*Salmonella sp.*, *E. coli*), fungi and antibiotic-resistant microbes interact with chemical contaminants such as heavy metals (lead, cadmium, arsenic) and pesticide residues (Fomina & Skorochod, 2020). The figure uses different arrow types to represent interactions: mutual influences, where microbes and chemicals affect each other's behaviour or toxicity; degradation, where microbes alter or degrade contaminants; and bioavailability influences, where chemicals impact microbial growth or survival (Aralappanavar et al., 2024). Also, it outlines potential health risk pathways, such as cumulative toxicity, nutrient absorption disruption, and gut microbiome dysbiosis, alongside proposed areas for future research, including the role of fungal toxins, heavy metal bioavailability, and antibiotic resistance transfer mechanisms (Teffera et al., 2024). These proposed research gaps indicate critical areas for future investigation to ensure the safety of calabash chalk consumption (Table 9).

**Table 9:** Proposed research areas for calabash chalk safety

Research gaps	Description	Potential impact	References
Region-specific toxic profiles	Map regional variations in contaminant levels in calabash chalk	Develop region-based safety guidelines	Simonnet-Laprade et al. (2021)
Analysis of rare trace elements	Investigate less-studied elements such as thallium and antimony	Provide a more comprehensive risk profile	Gasull et al. (2024)
Interactive risks of chemical and microbial contaminants	Study how contaminants interact to affect health risks	Improve accuracy of risk assessments	Espinosa et al. (2020)
Safe thresholds for heavy metals & pesticides	Set specific contaminant limits for geophagic soils	Establish clearer consumption guidelines	Emurotu and Onianwa, (2017)
Testing framework for calabash chalk Safety	Develop a comprehensive testing protocol specific to geophagic soils	Standardize safety evaluations for calabash chalk	Emurotu and Onianwa, (2017)

### Microbial diversity profiles

The potential risks of pathogenic microorganisms present in calabash chalk has been well established (Frazzoli et al., 2016; Kutalek et al., 2010). There is a need for further research to assess the full spectrum of pathogens present in calabash chalk especially for emerging pathogens. A comprehensive microbial profiling could provide insights into the types and prevalence of emerging pathogens present in different regions. There is also need to understand the specific impact of pathogen transmission from calabash chalk on pregnancy outcomes, including the risk of miscarriage, preterm birth, or foetal development defects (Wei et al., 2021). In addition, research into effective decontamination methods, such as sterilization techniques or the use of natural antimicrobial agents, could help make calabash chalk consumption safer. Similarly, limited research exist on how various pathogens within calabash chalk might survive under different storage and environmental conditions. Studying the heat resistance and overall stability of pathogens in geophagic soils could help understand how long these organisms remain viable in calabash chalk consumed by pregnant women. This could lead to recommendations on storage practices and possible pasteurization techniques to reduce microbial risks (Xiao et al., 2022).

While there is evidence of beneficial probiotic strains such as *Lactobacillus sp.* and *Bifidobacterium sp.*, the presence of pathogenic microorganisms or contaminants, including *E. coli, Salmonella sp.* and fungi such as *Candida sp.*, presents significant health risks. However, the balance within this community of microbes is largely unknown. Research into this balance of beneficial and harmful microorganisms in calabash chalk could shed light on its actual probiotic potential (Ma et al., 2023). This would involve identifying any strains of beneficial microbes and assessing their concentrations, stability, and potential health benefits versus the risks posed by pathogens. This area could be particularly novel, as it explores the dual nature of microbial presence in calabash chalk, looking beyond pathogens alone (Stevens et al., 2021).Monitoring and regulating the microbial diversity of calabash chalk is essential for maximizing its probiotic potential while minimizing associated health risks (Aleman & Yadav, 2023; Stevens et al., 2021). Table 10 provides a summary of studies focused on the microbial diversity of calabash chalk in various regions and its potential to support gut health and immune function.

**Table 10:** Comparison of probiotic vs. pathogenic microflora balance in calabash chalk from different regions

Region	Sample size	Probiotic microflora	Pathogenic microflora	Microbial balance/findings	Potential health implications	References
Northern Nigeria	10 samples	*Lactobacillus sp., Bifidobacterium sp., Bacillus sp.*	*Salmonella sp., Escherichia coli, Clostridium perfringens*	The probiotic species were dominant in 60% of the samples, with low levels of pathogens in the remaining 40%. No significant pathogenic growth observed in samples from local communities where calabash chalk was traditionally consumed	The presence of *Lactobacillus sp.* and *Bifidobacterium sp.* suggests potential gut health benefits. Reduced pathogen load implies a naturally occurring protective effect. Potential for probiotic development from regional calabash chalk samples	Luise et al. (2022)
Southern Nigeria	15 samples	Lactic acid bacteria *(LAB), Saccharomyces sp.*	*Staphylococcus aureus, Listeria monocytogenes*	Probiotic microorganisms were present in all samples, but 30% contained moderate to high levels of *Staphylococcus sp.* and *Listeria sp.*. Geophagic practices may involve environmental contamination from local hygiene	LAB could contribute to the prevention of gastrointestinal disorders, while the presence of *Listeria sp.* suggests a need for hygiene improvements in calabash chalk handling. Further studies on probiotic potential needed (Alice Njolke Mafe et al., 2025)	Dufailu et al. (2021)
West Africa (Ghana)	12 samples	*Lactobacillus acidophilus, Enterococcus faecium*	*Salmonella enterica, Shigella sp.*	75% of samples showed a healthy probiotic profile, while 25% had detectable levels of pathogens. Probiotic bacteria, such as *Lactobacillus sp.*, dominated even in slightly contaminated samples	Natural probiotic communities in calabash chalk may help protect against infections and boost immunity. Regions with minimal contamination could serve as models for probiotic calabash chalk consumption practices	Khushboo et al. (2023)
East Africa (Kenya)	8 samples	*Lactobacillus sp., Streptococcus thermophilus*	*Campylobacter jejuni, Enteropathogenic E. coli*	Probiotic microbes were consistently found in all samples, with pathogens in trace amounts in 40% of the samples. High-quality soil and proper handling contributed to lower pathogen prevalence	Probiotic bacteria may offer health benefits in preventing foodborne diseases. The ability of *Streptococcus sp.* and *Lactobacillus sp.* to suppress pathogenic bacteria supports the potential therapeutic properties of calabash chalk in this region	Webale et al. (2020)
Central Africa (Cameroon)	9 samples	*Bifidobacterium longum, Enterococcus faecalis*	*Pseudomonas aeruginosa, Vibrio cholerae*	High levels of *Bifidobacterium sp.* and *Enterococcus* species were noted, but 20% of the samples had contamination from waterborne pathogens, especially Vibrio and Pseudomonas	Probiotic strains may help in balancing gut microbiota (Alice N. Mafe & Büsselberg, 2025a), potentially improving digestion and immunity. In regions with water contamination, natural probiotic content could help mitigate disease transmission risks	Founou et al. (2024)

Advancements in omics technologies offer a novel approach to studying the microbial communities of calabash chalk. Traditional culturing methods will not give the detailed information about the diversity and composition of microorganisms in calabash chalk, but omics technology can provide a comprehensive snapshot of the microbial landscape (Lema et al., 2023). The use of high-throughput sequencing technologies to analyse the microbial communities of calabash chalk can reveal previously unknown species and their functional potentials, which will allow for a more complete insight of the microbial dynamics in geophagic soils (Jo et al., 2020). In addition to identifying microbial species, functional genomics can be used to explore the specific genes and pathways involved in pathogen resistance, antimicrobial production, and nutrient metabolism in calabash chalk. This could lead to the discovery of novel enzymes or metabolic pathways with potential applications in biotechnology or healthcare (Rabapane et al., 2022).

## Current regulations and safety standards on calabash chalk consumption

Calabash chalk consumption, commonly referred to as geophagy, is a practice that dates back to ancient times and persists across various cultures worldwide. While historically associated with traditional beliefs, medicinal uses, and dietary supplementation (Mafe & Büsselberg, 2025a, 2025b), modern science has raised concerns regarding its safety and potential health risks. The presence of contaminants such as heavy metals, pesticides, and pathogenic microorganisms in the soil can pose significant health hazards, emphasizing the need for stringent regulations and safety standards.

Regulatory frameworks and safety guidelines governing soil consumption are vital to protecting public health and addressing potential risks. These standards vary across regions and are informed by research in toxicology, microbiology, and environmental science. They aim to establish permissible limits for contaminants, identify safe sources of soil, and promote public awareness of the associated health risks. This document outlines the current regulations and safety measures designed to manage the practice of soil consumption while ensuring it is approached with caution in both traditional and modern contexts (Braine et al., 2024).

### International standards for soil consumption safety

The consumption of soil, including calabash chalk, as a traditional practice, especially among pregnant women, raises significant concerns regarding its safety and the associated health risks. While geophagia is prevalent in various cultures, especially in regions of Africa, international regulatory frameworks often overlook specific guidelines for soil consumption. Current safety standards primarily focus on contaminants such as heavy metals, pesticides, and microorganisms, but few explicitly address the safety of soils consumed by humans (Narh et al., 2021). It is critical to understand how international standards for contaminants in consumable products can apply to soils consumed in geophagic practices and whether existing regulations are sufficient to protect consumers, particularly vulnerable populations such as pregnant women. Table 11 compares international safety standards for contaminants, specifically focusing on acceptable limits for heavy metals and pesticides in soils, with a note on their relevance to geophagic practices. Current safety standards for soil contaminants are primarily designed for agricultural or residential use, not for direct human consumption, which leaves most geophagic soils non-compliant with these standards (Lar et al., 2015). This oversight poses potential health risks, as geophagic practices involve the direct ingestion of soil that may contain even trace amounts of contaminants such as lead (Pb), cadmium (Cd), and arsenic (As). Such contaminants, when ingested regularly, can accumulate in the body and lead to cumulative toxicity, potentially at levels far exceeding those encountered through agricultural produce (Das et al., 2023). Therefore, this comparison highlights critical gaps in existing safety standards when applied to geophagy, underscoring the need to assess and potentially adjust contaminant thresholds for soils consumed in this unique manner (Oyebanjo et al., 2020).

**Table 11:** Toxicological reports of heavy metals and pesticide residues detected in edible soils and calabash chalk samples

Sample type	Contaminants detected	Concentration range	Reported health implications	Study reference/DOI
Calabash chalk	Lead (Pb), Arsenic (As), Cadmium (Cd), Mercury (Hg)	Pb: 50–95 mg/kg, Cd: 1.8–3.0 mg/kg, As: 7.5 mg/kg	Neurotoxicity, kidney dysfunction, anemia, developmental disorders	Akpantah et al. (2010)
Calabash chalk	Lead (Pb), Arsenic (As), DDT residues	Pb: 65.2 mg/kg, DDT: 0.07 mg/kg	Impairs locomotor activities and social behaviour	Owhorji et al. (2019)
White clay (geophagic sample, Ghana)	Mercury (Hg), Lead (Pb), Chlorpyrifos (pesticide)	Hg: 0.45 mg/kg, Pb: 82.3 mg/kg	Neurodevelopmental harm, immunotoxicity, pesticide-induced oxidative stress	Kortei et al. (2020)
Amazonian forest soil (geophagic clay)	Mercury (Hg), DDT, Arsenic (As)	Hg: 1.1 mg/kg, DDT: 0.09 mg/kg	Developmental neurotoxicity, immune modulation, cancer risk	Lar et al. (2015)

### Recommendations for safety and regulatory gaps

The consumption of calabash chalk and similar geophagic substances presents unique health challenges due to potential contamination with heavy metals, pesticides, and microbial pathogens. Current safety standards and regulatory frameworks largely overlook these risks, as they are primarily designed for agricultural or residential soil rather than for direct ingestion (Perković et al., 2022). Handling these gaps requires specific guidelines and comprehensive testing protocols to ensure the safety of geophagic practices.

Current soil contaminant standards have primarily been developed for agricultural or residential use rather than direct human consumption. This focus means that the standards in place do not adequately account for soils intended for geophagic use, leaving most geophagic soils non-compliant with these established guidelines. Without adjustments, existing regulations may overlook the unique exposure risks associated with direct soil consumption, particularly in regions where geophagy is common (Amiri et al., 2022). Geophagy, the practice of consuming soil, can expose individuals to trace contaminants such as lead (Pb), cadmium (Cd), and arsenic (As). When ingested regularly, even small amounts of these contaminants can accumulate in the body, leading to cumulative toxicity (Olajide-Kayode et al., 2023). This risk is particularly concerning for geophagic soils, as their contaminant levels can exceed those typically found in agricultural products, presenting heightened health risks for consumers who ingest soil more frequently (Olisa et al., 2023).This assessment highlights significant gaps in current safety standards when applied to the unique context of geophagy. To better protect public health, there is a pressing need to evaluate and potentially adjust contaminant thresholds specifically for soils that may be consumed directly. Revising these standards would help address the unique risks associated with geophagy and ensure that regulations are inclusive of all forms of soil use.

**Fig. 3:**
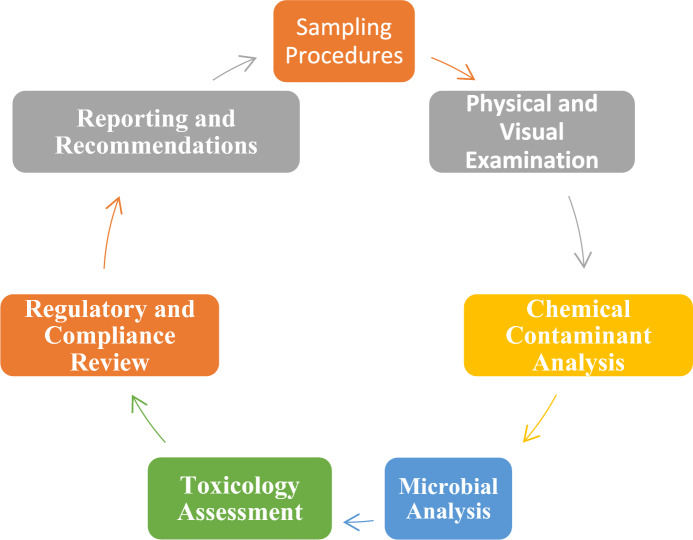
Testing framework for calabash chalk

A proposed testing framework (Fig. 3) for calabash chalk provides a comprehensive and systematic approach for evaluating the safety and quality of calabash chalk, a type of edible clay consumed in geophagic practices. This framework begins with sampling procedures, emphasizing the importance of random sampling to ensure representativeness. Samples are collected from diverse sources, such as market stalls and various geographic regions, and stored in airtight, contamination-free containers to preserve their chemical and microbial integrity. Detailed documentation, including source information, collection dates, and environmental conditions, is essential to contextualize the findings accurately.

The next step, physical and visual examination, involves inspecting the samples for visible contaminants, irregularities, and physical properties such as moisture content, particle size, and density, as these factors significantly influence microbial growth and contaminant absorption. This is followed by chemical contaminant analysis, which includes testing for heavy metals (e.g., lead, cadmium, mercury, arsenic) using advanced techniques such as atomic absorption spectroscopy (AAS) or inductively coupled plasma mass spectrometry (ICP-MS). The analysis also involves screening for pesticide residues, which may persist from soil or storage environments, and evaluating the elemental composition to balance beneficial minerals (e.g., calcium, magnesium, iron) against harmful ones (Sarkar et al., 2022).

Microbial analysis constitutes another critical component of the framework. This step involves pathogen screening for bacterial contaminants such as *Salmonella sp.*, *Escherichia coli* and *Staphylococcus aureus*, using both culture-based methods and rapid microbial detection systems. Fungal and yeast testing identifies mycotoxin-producing moulds and fungi, while antibiotic resistance testing evaluates microbial contaminants for resistance markers, which present significant health risks if pathogenic bacteria are ingested (Kabiraz et al., 2023).

The framework then proceeds to toxicology assessment, where acute and chronic toxicity tests are conducted to determine the health risks from prolonged exposure to contaminants such as heavy metals and pesticides. Bioavailability testing assesses how much of these harmful substances are absorbed during ingestion, providing a realistic estimation of the exposure risk to consumers (Kalyabina et al., 2021).

The final stages include regulatory and compliance review, where findings are compared to existing safety standards from organizations such as WHO and FAO. However, the framework acknowledges that most standards focus on agricultural or residential soil rather than edible clays such as calabash chalk, underscoring the need for geophagy-specific guidelines.

The framework emphasizes reporting and recommendations, summarizing findings in a detailed risk assessment report. The report includes guidance on safe consumption levels (where possible) and best storage practices to minimize contamination risks. Critical gaps in current safety protocols are also highlighted, such as the lack of geophagy-specific guidelines, limited pesticide and heavy metal standards for edible clays, and inadequate screening for microbial contaminants, particularly antibiotic-resistant pathogens (Krewski, 2022).

This comprehensive framework not only provides a roadmap for ensuring the safety of calabash chalk but also calls attention to the critical areas requiring further research and regulatory intervention.

## Conclusion

The consumption of calabash chalk presents both potential health risks and benefits, but significant regulatory gaps remain in ensuring its safety. While calabash chalk may offer some nutritional or cultural benefits, the health risks associated with its consumption such as exposure to heavy metals (lead, cadmium, arsenic), pesticide residues, and harmful microbes pose serious concerns, especially with long-term use especially to maternal health. These contaminants can lead to cumulative toxicity, affecting various organs and systems, including the gastrointestinal, renal, and neurological systems. Furthermore, current regulatory frameworks primarily focus on soil for agricultural or residential use, leaving geophagic soils such as calabash chalk largely non-compliant. The lack of specific safety standards for calabash chalk highlights the need for tailored guidelines and research to address the unique risks posed by its consumption to safeguard public health and harness the huge therapeutic potential derived from its consumption. However, it is important to note that toxicological studies specifically examining calabash chalk are currently limited. This gap in the literature underscores an urgent call for robust, targeted toxicological investigations to validate suspected risks, establish safe thresholds, and guide evidence-based regulation and public awareness.

## Data Availability

No datasets were generated or analysed during the current study.

## References

[CR1] Abdelhamid, A. G., El-Masry, S. S., & El-Dougdoug, N. K. (2019). Probiotic Lactobacillus and Bifidobacterium strains possess safety characteristics, antiviral activities and host adherence factors revealed by genome mining. *EPMA Journal,**10*(4), 337–350. 10.1007/s13167-019-00184-z 31832110 10.1007/s13167-019-00184-z PMC6883010

[CR2] Abrahams, P. W., Davies, T. C., Solomon, A. O., Trow, A. J., & Wragg, J. (2013). Human Geophagia, calabash chalk and undongo: Mineral element nutritional implications. *PLoS ONE,**8*(1), Article e53304. 10.1371/journal.pone.0053304 23308189 10.1371/journal.pone.0053304 PMC3538771

[CR3] Abrahams, P. W., & Parsons, J. A. (1997). Geophagy in the tropics: An appraisal of three geophagical materials. *Environmental Geochemistry and Health*, 19–22.

[CR4] da Acioly, T. M., da Silva, M. F., Barbosa, L. A., Iannacone, J., & Viana, D. C. (2024). Levels of potentially toxic and essential elements in water and estimation of human health risks in a river located at the interface of Brazilian Savanna and Amazon Biomes (Tocantins River). *Toxics,**12*(7), 444. 10.3390/toxics12070444 39058096 10.3390/toxics12070444 PMC11280896

[CR5] Adil, N., Ashraf, K., Munir, M., Mohiuddin, M., Abbasi, A., Riaz, U., et al. (2023). Pesticides, heavy metals and plasticizers: Contamination and risk assessment of drinking-water quality. *Sustainability,**15*(17), 13263. 10.3390/su151713263

[CR6] Agoro, M. A., Adeniji, A. O., Adefisoye, M. A., & Okoh, O. O. (2020). Heavy metals in wastewater and sewage sludge from selected municipal treatment plants in Eastern Cape Province South Africa. *Water,**12*(10), 2746. 10.3390/w12102746

[CR7] Ahmad, M. F., Ahmad, F. A., Alsayegh, A. A., Zeyaullah, M., AlShahrani, A. M., Muzammil, K., et al. (2024). Pesticides impacts on human health and the environment with their mechanisms of action and possible countermeasures. *Heliyon,**10*(7), Article e29128. 10.1016/j.heliyon.2024.e29128 38623208 10.1016/j.heliyon.2024.e29128 PMC11016626

[CR8] Ahmed, S., Siddique, M. A., Rahman, M., Bari, M. L., & Ferdousi, S. (2019). A study on the prevalence of heavy metals, pesticides, and microbial contaminants and antibiotics resistance pathogens in raw salad vegetables sold in Dhaka Bangladesh. *Heliyon,**5*(2), Article e01205. 10.1016/j.heliyon.2019.e01205 30805565 10.1016/j.heliyon.2019.e01205 PMC6374544

[CR9] Akpantah, A. O., Ibok, O. S., Ekong, M. B., Eluwa, M. A., & Bassey Ekanem, T. (2010). The effect of calabash chalk on some hematological parameters in female adult Wistar rats. *Turkish Journal of Hematology,**27*(3), 177–181. 10.5152/tjh.2010.25 27263602 10.5152/tjh.2010.25

[CR10] Alelign, T., Chalchisa, D., Fekadu, N., Solomon, D., Sisay, T., Debella, A., & Petros, B. (2020). Evaluation of acute and sub-acute toxicity of selected traditional antiurolithiatic medicinal plant extracts in Wistar albino rats. *Toxicology Reports,**7*, 1356–1365. 10.1016/j.toxrep.2020.10.001 33102139 10.1016/j.toxrep.2020.10.001 PMC7569265

[CR11] Aleman, R. S., & Yadav, A. (2023). Systematic review of probiotics and their potential for developing functional nondairy foods. *Applied Microbiology,**4*(1), 47–69. 10.3390/applmicrobiol4010004

[CR12] Alengebawy, A., Abdelkhalek, S. T., Qureshi, S. R., & Wang, M.-Q. (2021). Heavy metals and pesticides toxicity in agricultural soil and plants: Ecological risks and human health implications. *Toxics,**9*(3), 42. 10.3390/toxics9030042 33668829 10.3390/toxics9030042 PMC7996329

[CR13] Aleruchi, O., Ogbonna, S. I., Amadi, E., & Nnodim, L. C. (2022). Microbiological assessment and antimicrobial properties of edible clay (Nzu) sold in port harcourt metropolis. *Acta Scientific Microbiology (ISSN: 2581–3226)*, *5*(9).

[CR14] Algharably, E. A., Di Consiglio, E., Testai, E., Pistollato, F., Bal-Price, A., Najjar, A., et al. (2023). Prediction of in vivo prenatal chlorpyrifos exposure leading to developmental neurotoxicity in humans based on in vitro toxicity data by quantitative in vitro–in vivo extrapolation. *Frontiers in Pharmacology,**14*, Article 1136174. 10.3389/fphar.2023.1136174 36959852 10.3389/fphar.2023.1136174 PMC10027916

[CR15] Alvarado-Esquivel, C., Pacheco-Vega, S. J., Salcedo-Jaquez, M., Sánchez-Anguiano, L. F., Hernández-Tinoco, J., Rábago-Sánchez, E., et al. (2015). Stillbirth history and Toxoplasma gondii infection in women attending public health centers in a northern Mexican city. *European Journal of Microbiology and Immunology,**5*(2), 164–171. 10.1556/1886.2015.00009 26185685 10.1556/1886.2015.00009 PMC4500068

[CR16] Amiri, H., Hoseini, M., Abbasi, S., Malakootian, M., Hashemi, M., Jaafarzadeh, N., & Turner, A. (2022). Geophagy and microplastic ingestion. *Journal of Food Composition and Analysis,**106*, Article 104290. 10.1016/j.jfca.2021.104290

[CR17] Ansari, I., El-Kady, M. M., El Din Mahmoud, A., Arora, C., Verma, A., Rajarathinam, R., et al. (2024). Persistent pesticides: Accumulation, health risk assessment, management and remediation: An overview. *Desalination and Water Treatment,**317*, Article 100274. 10.1016/j.dwt.2024.100274

[CR18] Aprioku, J., & Ogwo-Ude, E. (2018). Gestational toxicity of Calabash chalk (Nzu) in Wistar rats. *International Journal of Applied and Basic Medical Research,**8*(4), 249. 10.4103/ijabmr.IJABMR_412_17 30598913 10.4103/ijabmr.IJABMR_412_17 PMC6259302

[CR19] Aralappanavar, V. K., Mukhopadhyay, R., Yu, Y., Liu, J., Bhatnagar, A., Praveena, S. M., et al. (2024). Effects of microplastics on soil microorganisms and microbial functions in nutrients and carbon cycling – A review. *Science of the Total Environment,**924*, Article 171435. 10.1016/j.scitotenv.2024.171435 38438042 10.1016/j.scitotenv.2024.171435

[CR20] Auer, L., Lazuka, A., Sillam-Dussès, D., Miambi, E., O’Donohue, M., & Hernandez-Raquet, G. (2017). Uncovering the potential of termite gut microbiome for lignocellulose bioconversion in anaerobic batch bioreactors. *Frontiers in Microbiology,**8*, 2623. 10.3389/fmicb.2017.02623 29312279 10.3389/fmicb.2017.02623 PMC5744482

[CR21] Aynalem, B. Y., Melesse, M. F., & Bitewa, Y. B. (2023). Cultural beliefs and traditional practices during pregnancy, child birth, and the postpartum period in East Gojjam Zone, Northwest Ethiopia: A qualitative study. *Women’s Health Reports,**4*(1), 415–422. 10.1089/whr.2023.0024 10.1089/whr.2023.0024 PMC10460962 37645589

[CR22] Azmi, N. N., Mahyudin, N. A., Wan Omar, W. H., Mahmud Ab Rashid, N.-K., Ishak, C. F., Abdullah, A. H., & Sharples, G. J. (2021). Antibacterial activity of clay soils against food-borne salmonella typhimurium and staphylococcus aureus. *Molecules,**27*(1), 170. 10.3390/molecules27010170 35011396 10.3390/molecules27010170 PMC8746575

[CR23] Babah, O. A., Akinajo, O. R., Beňová, L., Hanson, C., Abioye, A. I., Adaramoye, V. O., et al. (2024). Prevalence of and risk factors for iron deficiency among pregnant women with moderate or severe anaemia in Nigeria: A cross-sectional study. *BMC Pregnancy and Childbirth,**24*(1), 39. 10.1186/s12884-023-06169-1 38182997 10.1186/s12884-023-06169-1 PMC10768359

[CR24] Bæk, O., Sangild, P. T., Thymann, T., & Nguyen, D. N. (2019). Growth restriction and systemic immune development in preterm piglets. *Frontiers in Immunology,**10*, 2402. 10.3389/fimmu.2019.02402 31649685 10.3389/fimmu.2019.02402 PMC6795705

[CR25] Behroozian, S., Svensson, S. L., Li, L. Y., & Davies, J. E. (2020). Broad-spectrum antimicrobial and antibiofilm activity of a natural clay mineral from British Columbia Canada. *mBio,**11*(5), 10. 10.1128/mBio.02350-20 10.1128/mBio.02350-20 PMC7542368 33024043

[CR26] Bisi-Johnson, M. A., Oyelade, H. A., Adediran, K. A., & Akinola, S. A. (2013). Microbial evaluation of geophagic and cosmetic clays from southern and western Nigeria: Potential natural nanomaterials. *International Journal of Environmental Chemical, Ecological, Geological and Geophysical Engineering,**7*(12), 832–835.

[CR27] Bonaterra, A., Badosa, E., Daranas, N., Francés, J., Roselló, G., & Montesinos, E. (2022). Bacteria as biological control agents of plant diseases. *Microorganisms,**10*(9), 1759. 10.3390/microorganisms10091759 36144361 10.3390/microorganisms10091759 PMC9502092

[CR28] Bonglaisin, J. N., Kunsoan, N. B., Bonny, P., Matchawe, C., Tata, B. N., Nkeunen, G., & Mbofung, C. M. (2022). Geophagia: Benefits and potential toxicity to human—A review. *Frontiers in Public Health,**10*, Article 893831. 10.3389/fpubh.2022.893831 35958861 10.3389/fpubh.2022.893831 PMC9360771

[CR29] Braine, M. F., Kearnes, M., & Khan, S. J. (2024). Quality and risk management frameworks for biosolids: An assessment of current international practice. *Science of the Total Environment,**915*, Article 169953. 10.1016/j.scitotenv.2024.169953 38215849 10.1016/j.scitotenv.2024.169953

[CR30] Caillet, P., Poirier, M., Grall-Bronnec, M., Marchal, E., Pineau, A., Pintas, C., et al. (2019). High prevalence of kaolin consumption in migrant women living in a major urban area of France: A cross-sectional investigation. *PLoS ONE,**14*(7), Article e0220557. 10.1371/journal.pone.0220557 31365572 10.1371/journal.pone.0220557 PMC6668907

[CR31] Caldrer, S., Ursini, T., Santucci, B., Motta, L., & Angheben, A. (2022). Soil-transmitted helminths and anaemia: A neglected association outside the tropics. *Microorganisms,**10*(5), 1027. 10.3390/microorganisms10051027 35630469 10.3390/microorganisms10051027 PMC9143297

[CR32] Castrejón-Godínez, M. L., Tovar-Sánchez, E., Valencia-Cuevas, L., Rosas-Ramírez, M. E., Rodríguez, A., & Mussali-Galante, P. (2021). Glyphosate pollution treatment and microbial degradation alternatives, A review. *Microorganisms,**9*(11), 2322. 10.3390/microorganisms9112322 34835448 10.3390/microorganisms9112322 PMC8625783

[CR33] Cavalier, H., Trasande, L., & Porta, M. (2023). Exposures to pesticides and risk of cancer: Evaluation of recent epidemiological evidence in humans and paths forward. *International Journal of Cancer,**152*(5), 879–912. 10.1002/ijc.34300 36134639 10.1002/ijc.34300 PMC9880902

[CR34] Chan, M. Y., & Smith, M. A. (2018). Infections in pregnancy. *Comprehensive toxicology* (pp. 232–249). Elsevier.

[CR35] Chinko, B. C., Amah-Tariah, F. S., Ekenna, I. C., & Okpa, O. A. (2022). Evaluation of the effects of calabash chalk on the haematological profile of Wistar rats. *Notulae Scientia Biologicae,**14*(3), 11281. 10.55779/nsb14311281

[CR36] Chisanga, K., Mbega, E. R., & Ndakidemi, P. A. (2020). Prospects of using termite mound soil organic Amendment for enhancing soil nutrition in&#13 Southern Africa. *Plants,**9*(5), 649. 10.3390/plants9050649 32443902 10.3390/plants9050649 PMC7284692

[CR37] Chrustek, A., Hołyńska-Iwan, I., Dziembowska, I., Bogusiewicz, J., Wróblewski, M., Cwynar, A., & Olszewska-Słonina, D. (2018). Current research on the safety of pyrethroids used as insecticides. *Medicina,**54*(4), 61. 10.3390/medicina54040061 30344292 10.3390/medicina54040061 PMC6174339

[CR38] Cuesta, E. B., Coulibaly, B., Bukhari, T., Eiglmeier, K., Kone, R., Coulibaly, M. B., et al. (2021). Comprehensive ecological and geographic characterization of eukaryotic and prokaryotic microbiomes in African anopheles. *Frontiers in Microbiology,**12*, Article 635772. 10.3389/fmicb.2021.635772 34054746 10.3389/fmicb.2021.635772 PMC8153677

[CR39] Dack, K., Fell, M., Taylor, C. M., Havdahl, A., & Lewis, S. J. (2022). Prenatal mercury exposure and neurodevelopment up to the age of 5 years: A systematic review. *International Journal of Environmental Research and Public Health,**19*(4), 1976. 10.3390/ijerph19041976 35206164 10.3390/ijerph19041976 PMC8871549

[CR40] Das, S., Sultana, K. W., Ndhlala, A. R., Mondal, M., & Chandra, I. (2023). Heavy metal pollution in the environment and its impact on health: Exploring green technology for remediation. *Environmental Health Insights*. 10.1177/11786302231201259 10.1177/11786302231201259 PMC10559720 37808962

[CR41] Daskalakis, G., Psarris, A., Koutras, A., Fasoulakis, Z., Prokopakis, I., Varthaliti, A., et al. (2023). Maternal infection and preterm birth: From molecular basis to clinical implications. *Children,**10*(5), 907. 10.3390/children10050907 37238455 10.3390/children10050907 PMC10217143

[CR42] Davies, T. C. (2023). Current status of research and gaps in knowledge of geophagic practices in Africa. *Frontiers in Nutrition,**9*, 1084589. 10.3389/fnut.2022.1084589 36890865 10.3389/fnut.2022.1084589 PMC9987423

[CR43] Dean, J., Deary, M., Gbefa, B., & Scott, W. (2004). Characterisation and analysis of persistent organic pollutants and major, minor and trace elements in Calabash chalk. *Chemosphere,**57*(1), 21–25. 10.1016/j.chemosphere.2004.05.023 15288195 10.1016/j.chemosphere.2004.05.023

[CR44] Dufailu, O. A., Yaqub, M. O., Owusu-Kwarteng, J., & Addy, F. (2021). Prevalence and characteristics of Listeria species from selected African countries. *Tropical Diseases, Travel Medicine and Vaccines,**7*(1), 26. 10.1186/s40794-021-00151-5 34521480 10.1186/s40794-021-00151-5 PMC8442394

[CR45] Ekong, M. B., Andrioli, A., Israel, I. E., Ifot, E. I., Dickson, S. E., Scambi, I., et al. (2024). Evaluation of prenatal calabash chalk geophagy on the developing brain of Wistar rats. *IBRO Neuroscience Reports,**16*, 443–454. 38544793 10.1016/j.ibneur.2024.03.007 PMC10966185

[CR46] Ekong, M. B., Peter, A. I., Ekanem, T. B., & Osim, E. E. (2015). Determination of elemental composition and median lethal dose of calabash chalk. *Int J Biol Med Res,**6*(2), 4902–4906.

[CR47] El Hayek, P., Boueri, M., Nasr, L., Aoun, C., Sayad, E., & Jallad, K. (2023). Cholera infection risks and cholera vaccine safety in pregnancy. *Infectious Diseases in Obstetrics and Gynecology,**2023*, 1–8. 10.1155/2023/4563797 10.1155/2023/4563797 PMC10228220 37260611

[CR48] Ellwanger, J. H., & Chies, J. A. B. (2023). Brazil’s heavy metal pollution harms humans and ecosystems. *Science in One Health,**2*, Article 100019. 10.1016/j.soh.2023.100019 39077034 10.1016/j.soh.2023.100019 PMC11262263

[CR49] Emurotu, J. E., & Onianwa, P. C. (2017). Bioaccumulation of heavy metals in soil and selected food crops cultivated in Kogi State, north central Nigeria. *Environmental Systems Research,**6*(1), 21. 10.1186/s40068-017-0098-1

[CR50] Espinosa, M. F., Sancho, A. N., Mendoza, L. M., Mota, C. R., & Verbyla, M. E. (2020). Systematic review and meta-analysis of time-temperature pathogen inactivation. *International Journal of Hygiene and Environmental Health,**230*, Article 113595. 10.1016/j.ijheh.2020.113595 32814236 10.1016/j.ijheh.2020.113595

[CR51] Fomina, M., & Skorochod, I. (2020). Microbial interaction with clay minerals and its environmental and biotechnological implications. *Minerals,**10*(10), 861. 10.3390/min10100861

[CR52] Ford, J. D., King, N., Galappaththi, E. K., Pearce, T., McDowell, G., & Harper, S. L. (2020). The resilience of indigenous peoples to environmental change. *One Earth,**2*(6), 532–543. 10.1016/j.oneear.2020.05.014

[CR53] Founou, R. C., Founou, L. L., Allam, M., Ismail, A., & Essack, S. Y. (2024). Genome analysis of multidrug resistant Enterococcus faecium and Enterococcus faecalis circulating among hospitalized patients in uMgungundlovu District, KwaZulu-Natal. *South Africa. BMC Infectious Diseases,**24*(1), 671. 10.1186/s12879-024-09380-3 38965470 10.1186/s12879-024-09380-3 PMC11225414

[CR54] Frazzoli, C., Pouokam, G. B., Mantovani, A., & Orisakwe, O. E. (2016). Health risks from lost awareness of cultural behaviours rooted in traditional medicine: An insight in geophagy and mineral intake. *Science of the Total Environment,**566–567*, 1465–1471. 10.1016/j.scitotenv.2016.06.028 10.1016/j.scitotenv.2016.06.028 27342642

[CR55] Gasull, M., Camargo, J., Pumarega, J., Henríquez-Hernández, L. A., Campi, L., Zumbado, M., et al. (2024). Blood concentrations of metals, essential trace elements, rare earth elements and other chemicals in the general adult population of Barcelona: Distribution and associated sociodemographic factors. *Science of the Total Environment,**909*, Article 168502. 10.1016/j.scitotenv.2023.168502 37977377 10.1016/j.scitotenv.2023.168502

[CR56] Genchi, G., Carocci, A., Lauria, G., Sinicropi, M. S., & Catalano, A. (2020a). Nickel: Human health and environmental toxicology. *International Journal of Environmental Research and Public Health,**17*(3), 679. 10.3390/ijerph17030679 31973020 10.3390/ijerph17030679 PMC7037090

[CR57] Genchi, G., Sinicropi, M. S., Lauria, G., Carocci, A., & Catalano, A. (2020b). The effects of cadmium toxicity. *International Journal of Environmental Research and Public Health,**17*(11), 3782. 10.3390/ijerph17113782 32466586 10.3390/ijerph17113782 PMC7312803

[CR58] Georgieff, M. K. (2020). Iron deficiency in pregnancy. *American Journal of Obstetrics and Gynecology,**223*(4), 516–524. 10.1016/j.ajog.2020.03.006 32184147 10.1016/j.ajog.2020.03.006 PMC7492370

[CR59] Grassi, A., Cafarchia, C., Decaro, N., Rhimi, W., De Laurentiis, V., D’Annunzio, G., et al. (2024). Systemic Candida infection and pulmonary aspergillosis in an alpaca (Vicugna pacos): A case report. *Journal of Fungi,**10*(3), 227. 10.3390/jof10030227 38535235 10.3390/jof10030227 PMC10971701

[CR60] Grenda, T., Jarosz, A., Sapała, M., Grenda, A., Patyra, E., & Kwiatek, K. (2023). Clostridium perfringens—Opportunistic foodborne pathogen. *Its Diversity and Epidemiological Significance. Pathogens,**12*(6), 768. 10.3390/pathogens12060768 37375458 10.3390/pathogens12060768 PMC10304509

[CR61] Guimarães, L. D. D., Lima, E. S. A., de Souza, C., da Pinheiro, H. S. K., & da Amaral Sobrinho, N. M. B. (2024). Spatial distribution and factors influencing the accumulation of toxic metals in soils in mountain agroecosystems, Rio De Janeiro Brazil. *Environmental Geochemistry and Health,**46*(10), 394. 10.1007/s10653-024-02175-0 39180674 10.1007/s10653-024-02175-0

[CR62] Gundacker, C., Forsthuber, M., Szigeti, T., Kakucs, R., Mustieles, V., Fernandez, M. F., et al. (2021). Lead (Pb) and neurodevelopment: A review on exposure and biomarkers of effect (BDNF, HDL) and susceptibility. *International Journal of Hygiene and Environmental Health,**238*, Article 113855. 10.1016/j.ijheh.2021.113855 34655857 10.1016/j.ijheh.2021.113855

[CR63] Gwinn, K. D., Leung, M. C. K., Stephens, A. B., & Punja, Z. K. (2023). Fungal and mycotoxin contaminants in cannabis and hemp flowers: Implications for consumer health and directions for further research. *Frontiers in Microbiology,**14*, Article 1278189. 10.3389/fmicb.2023.1278189 37928692 10.3389/fmicb.2023.1278189 PMC10620813

[CR64] Huebl, L., Leick, S., Guettl, L., Akello, G., & Kutalek, R. (2016). Geophagy in Northern Uganda: Perspectives from consumers and clinicians. *The American Society of Tropical Medicine and Hygiene,**95*(6), 1440–1449. 10.4269/ajtmh.15-0579 10.4269/ajtmh.15-0579 PMC5154465 27698274

[CR65] Iqbal, S., Klammer, N., & Ekmekcioglu, C. (2019). The effect of electrolytes on blood pressure: A brief summary of meta-analyses. *Nutrients,**11*(6), 1362. 10.3390/nu11061362 31212974 10.3390/nu11061362 PMC6627949

[CR66] Ismail, A., Sial, N., Rehman, R., Abid, S., & Ismail, M. S. (2023). Survival, growth, behavior, hematology and serum biochemistry of mice under different concentrations of orally administered amorphous silica nanoparticle. *Toxicology Reports,**10*, 659–668. 10.1016/j.toxrep.2023.05.006 37274627 10.1016/j.toxrep.2023.05.006 PMC10238806

[CR67] Izugbara, C. O. (2004). The cultural context of geophagy among pregnant and lactating Ngwa women of Southeastern Nigeria. *African Anthropologist,**10*(2), 180–199. 10.4314/aa.v10i2.23114

[CR68] Jo, J., Oh, J., & Park, C. (2020). Microbial community analysis using high-throughput sequencing technology: A beginner’s guide for microbiologists. *Journal of Microbiology,**58*(3), 176–192. 10.1007/s12275-020-9525-5 32108314 10.1007/s12275-020-9525-5

[CR69] Johan, P. D., Ahmed, O. H., Omar, L., & Hasbullah, N. A. (2021). Phosphorus transformation in soils following co-application of charcoal and wood ash. *Agronomy,**11*(10), 2010. 10.3390/agronomy11102010

[CR70] Kabiraz, M. P., Majumdar, P. R., Mahmud, M. M. C., Bhowmik, S., & Ali, A. (2023). Conventional and advanced detection techniques of foodborne pathogens: A comprehensive review. *Heliyon,**9*(4), Article e15482. 10.1016/j.heliyon.2023.e15482 37151686 10.1016/j.heliyon.2023.e15482 PMC10161726

[CR71] Kale, O. E., Awodele, O., & Akindele, A. J. (2019). Subacute and subchronic oral toxicity assessments of Acridocarpus smeathmannii (DC.) Guill. & Perr. root in Wistar rats. *Toxicology Reports,**6*, 161–175. 10.1016/j.toxrep.2019.01.005 30766799 10.1016/j.toxrep.2019.01.005 PMC6360914

[CR72] Kalyabina, V. P., Esimbekova, E. N., Kopylova, K. V., & Kratasyuk, V. A. (2021). Pesticides: Formulants, distribution pathways and effects on human health—a review. *Toxicology Reports,**8*, 1179–1192. 10.1016/j.toxrep.2021.06.004 34150527 10.1016/j.toxrep.2021.06.004 PMC8193068

[CR73] Kaur, R., Choudhary, D., Bali, S., Bandral, S. S., Singh, V., Ahmad, M. A., et al. (2024). Pesticides: An alarming detrimental to health and environment. *Science of the Total Environment,**915*, Article 170113. 10.1016/j.scitotenv.2024.170113 38232846 10.1016/j.scitotenv.2024.170113

[CR74] Khushboo, K. A., & Malik, T. (2023). Characterization and selection of probiotic lactic acid bacteria from different dietary sources for development of functional foods. *Frontiers in Microbiology,**14*, 1170725. 10.3389/fmicb.2023.1170725 37213505 10.3389/fmicb.2023.1170725 PMC10196247

[CR75] Kocyłowski, R., Lewicka, I., Grzesiak, M., Gaj, Z., Sobańska, A., Poznaniak, J., et al. (2018). Assessment of dietary intake and mineral status in pregnant women. *Archives of Gynecology and Obstetrics,**297*(6), 1433–1440. 10.1007/s00404-018-4744-2 29541858 10.1007/s00404-018-4744-2 PMC5945726

[CR76] Kortei, N. K., Koryo-Dabrah, A., Akonor, P. T., Manaphraim, N. Y. B., Ayim-Akonor, M., Boadi, N. O., et al. (2020). Potential health risk assessment of toxic metals contamination in clay eaten as pica (geophagia) among pregnant women of Ho in the Volta Region of Ghana. *BMC Pregnancy and Childbirth,**20*(1), 160. 10.1186/s12884-020-02857-4 32169034 10.1186/s12884-020-02857-4 PMC7071753

[CR77] Kotun, B., Adewara, O., Ayedun, J. S., Makinde, O. M., Oda, O., Adeleke, A. J., et al. (2024). Distribution, prevention and control of emerging food contaminations. In *Emerging Contaminants in Food and Food Products* (p. 304).

[CR78] Krewski, D. (2022). Development of an evidence-based risk assessment framework. *ALTEX.*10.14573/altex.2004041 10.14573/altex.2004041 PMC10080579 36098377

[CR79] Kumar, A., & Kaur, S. (2017). Calcium: A nutrient in pregnancy. *The Journal of Obstetrics and Gynecology of India,**67*(5), 313–318. 10.1007/s13224-017-1007-2 28867880 10.1007/s13224-017-1007-2 PMC5561751

[CR80] Kumar, M., Saadaoui, M., & Al Khodor, S. (2022). Infections and pregnancy: Effects on maternal and child health. *Frontiers in Cellular and Infection Microbiology,**12*, Article 873253. 10.3389/fcimb.2022.873253 35755838 10.3389/fcimb.2022.873253 PMC9217740

[CR81] Kutalek, R., Wewalka, G., Gundacker, C., Auer, H., Wilson, J., Haluza, D., et al. (2010). Geophagy and potential health implications: Geohelminths, microbes and heavy metals. *Transactions of the Royal Society of Tropical Medicine and Hygiene,**104*(12), 787–795. 10.1016/j.trstmh.2010.09.002 20889178 10.1016/j.trstmh.2010.09.002

[CR82] Kyei, N. N. A., Boakye, D., & Gabrysch, S. (2020). Maternal mycotoxin exposure and adverse pregnancy outcomes: A systematic review. *Mycotoxin Research,**36*(2), 243–255. 10.1007/s12550-019-00384-6 31989413 10.1007/s12550-019-00384-6 PMC7182542

[CR83] Lambraki, I. A., Chadag, M. V., Cousins, M., Graells, T., Léger, A., Henriksson, P. J. G., et al. (2023). Factors impacting antimicrobial resistance in the South East Asian food system and potential places to intervene: A participatory, one health study. *Frontiers in Microbiology,**13*, Article 992507. 10.3389/fmicb.2022.992507 36687632 10.3389/fmicb.2022.992507 PMC9849958

[CR84] Lar, U. A., Agene, J. I., & Umar, A. I. (2015). Geophagic clay materials from Nigeria: A potential source of heavy metals and human health implications in mostly women and children who practice it. *Environmental Geochemistry and Health,**37*(2), 363–375. 10.1007/s10653-014-9653-0 25416852 10.1007/s10653-014-9653-0

[CR85] Lema, N. K., Gemeda, M. T., & Woldesemayat, A. A. (2023). Recent advances in metagenomic approaches, applications, and challenges. *Current Microbiology,**80*(11), 347. 10.1007/s00284-023-03451-5 37733134 10.1007/s00284-023-03451-5

[CR86] Lete, I., & Alluέ, J. (2016). The effectiveness of ginger in the prevention of nausea and vomiting during pregnancy and chemotherapy. *Integrative Medicine Insights,**11*, IMI-S36273. 10.4137/IMI.S36273 10.4137/IMI.S36273 PMC4818021 27053918

[CR87] Luise, D., Bosi, P., Raff, L., Amatucci, L., Virdis, S., & Trevisi, P. (2022). Bacillus spp. probiotic strains as a potential tool for limiting the use of antibiotics, and improving the growth and health of pigs and chickens. *Frontiers in Microbiology,**13*, Article 801827. 10.3389/fmicb.2022.801827 35197953 10.3389/fmicb.2022.801827 PMC8859173

[CR88] Ma, L., Zhao, H., Wu, L. B., Cheng, Z., & Liu, C. (2023). Impact of the microbiome on human, animal, and environmental health from a One Health perspective. *Science in One Health,**2*, Article 100037. 10.1016/j.soh.2023.100037 39077043 10.1016/j.soh.2023.100037 PMC11262275

[CR89] Madziva, C., & Chinouya, M. J. (2023). African migrant women acquisition of clay for ingestion during pregnancy in London: A call for action. *Public Health,**223*, 110–116. 10.1016/j.puhe.2023.07.021 37634450 10.1016/j.puhe.2023.07.021

[CR90] Madziva, C., & Chinouya, M. J. (2020). Clay ingestion during pregnancy among black African Women in a North London Borough: Understanding cultural meanings, integrating indigenous and biomedical knowledge systems. *Frontiers in Sociology,**5*, 20. 10.3389/fsoc.2020.00020 33869429 10.3389/fsoc.2020.00020 PMC8022624

[CR91] Madziva, C., Chinouya, M. J., & Njoroge, K. (2024). Experiences of geophagy during pregnancy among African migrant women in London: Implications for public health interventions. *SSM - Qualitative Research in Health,**5*, Article 100431. 10.1016/j.ssmqr.2024.100431

[CR92] Mafe, A. N., & Büsselberg, D. (2024a). Mycotoxins in Food: Cancer Risks and Strategies for Control. *Foods,**13*(21), 3502. 10.3390/foods13213502 39517285 10.3390/foods13213502 PMC11545588

[CR93] Mafe, A. N., & Büsselberg, D. (2024b). Impact of metabolites from foodborne pathogens on cancer. *Foods,**13*(23), 3886. 10.3390/foods13233886 39682958 10.3390/foods13233886 PMC11640045

[CR94] Mafe, A. N., & Büsselberg, D. (2025a). Modulation of the neuro-cancer connection by metabolites of gut microbiota. *Biomolecules,**15*(2), 270. 10.3390/biom15020270 40001573 10.3390/biom15020270 PMC11853082

[CR95] Mafe, A. N., & Büsselberg, D. (2025b). Could a mediterranean diet modulate alzheimer’s disease progression? The role of gut microbiota and metabolite signatures in neurodegeneration. *Foods,**14*(9), 1559. 10.3390/foods14091559 40361641 10.3390/foods14091559 PMC12071848

[CR96] Mafe, A. N., Nkene, I. H., Ali, A. B. M., Edo, G. I., Akpoghelie, P. O., Yousif, E., et al. (2025). Smart probiotic solutions for mycotoxin mitigation: Innovations in food safety and sustainable agriculture. *Probiotics and Antimicrobial Proteins*. 10.1007/s12602-025-10569-4 10.1007/s12602-025-10569-4 40312537

[CR97] Mahamat, A. A., Obianyo, I. I., Ngayakamo, B., Bih, N. L., Ayeni, O., Azeko, S. T., & Savastano, H. (2021). Alkali activation of compacted termite mound soil for eco-friendly construction materials. *Heliyon,**7*(3), Article e06597. 10.1016/j.heliyon.2021.e06597 33869844 10.1016/j.heliyon.2021.e06597 PMC8035522

[CR98] Mahaney, W. C., Milner, M. W., Hs, M., Hancock, R. G. V., Aufreiter, S., Reich, M., & Wink, M. (2000). Mineral and chemical analyses of soils eaten by humans in Indonesia. *International Journal of Environmental Health Research,**10*(2), 93–109. 10.1080/09603120050021100

[CR99] Makinde, O. M., Ayeni, K. I., Sulyok, M., Krska, R., Adeleke, R. A., & Ezekiel, C. N. (2020). Microbiological safety of ready-to-eat foods in low- and middle-income countries: A comprehensive 10-year (2009 to 2018) review. *Comprehensive Reviews in Food Science and Food Safety,**19*(2), 703–732. 10.1111/1541-4337.12533 33325184 10.1111/1541-4337.12533

[CR100] Marín, R., Abad, C., Rojas, D., Chiarello, D. I., Rangel, H., Teppa-Garrán, A., et al. (2023). Magnesium salts in pregnancy. *Journal of Trace Elements and Minerals,**4*, Article 100071. 10.1016/j.jtemin.2023.100071

[CR101] Mate, A., Reyes-Goya, C., Santana-Garrido, Á., Sobrevia, L., & Vázquez, C. M. (2021). Impact of maternal nutrition in viral infections during pregnancy. *Biochimica et Biophysica Acta (BBA) Molecular Basis of Disease,**1867*(11), Article 166231. 10.1016/j.bbadis.2021.166231 34343638 10.1016/j.bbadis.2021.166231 PMC8325560

[CR102] Miller, J. D., Collins, S. M., Omotayo, M., Martin, S. L., Dickin, K. L., & Young, S. L. (2018). Geophagic earths consumed by women in western Kenya contain dangerous levels of lead, arsenic, and iron. *American Journal of Human Biology,**30*(4), E23130. 10.1002/ajhb.23130 29722093 10.1002/ajhb.23130 PMC6105564

[CR103] Mishra, R. K., Mentha, S. S., Misra, Y., & Dwivedi, N. (2023). Emerging pollutants of severe environmental concern in water and wastewater: A comprehensive review on current developments and future research. *Water-Energy Nexus,**6*, 74–95. 10.1016/j.wen.2023.08.002

[CR104] Moosavi, M. (2017). Bentonite clay as a natural remedy: A brief review. *Iranian Journal of Public Health,**46*(9), 1176–1183. 29026782 PMC5632318

[CR105] Mudonhi, N., & Nunu, W. N. (2022). Traditional medicine utilisation among pregnant women in Sub-saharan African countries: A systematic review of literature. *INQUIRY: The Journal of Health Care Organization, Provision, and Financing*. 10.1177/00469580221088618 10.1177/00469580221088618 PMC9073130 35506677

[CR106] Narh, C. T., Dzamalala, C. P., Mmbaga, B. T., Menya, D., Mlombe, Y., Finch, P., et al. (2021). Geophagia and risk of squamous cell esophageal cancer in the African esophageal cancer corridor: Findings from the <scp>ESCCAPE</scp> multicountry case-control studies. *International Journal of Cancer,**149*(6), 1274–1283. 10.1002/ijc.33688 34004024 10.1002/ijc.33688 PMC8411422

[CR107] Nasrabadi, M., Nazarian, M., Darroudi, M., Marouzi, S., Harifi-Mood, M. S., Samarghandian, S., & Farkhondeh, T. (2024). Carbamate compounds induced toxic effects by affecting Nrf2 signaling pathways. *Toxicology Reports,**12*, 148–157. 10.1016/j.toxrep.2023.12.004 38304697 10.1016/j.toxrep.2023.12.004 PMC10831123

[CR108] Neelotpol, S., Rezwan, R., & Shahriar, M. (2023). Chemical analysis of calabash chalk and its effect on locomotor activities and behavior in Swiss albino mice. *Heliyon,**9*(3), Article e14463. 10.1016/j.heliyon.2023.e14463 36994387 10.1016/j.heliyon.2023.e14463 PMC10040505

[CR109] Neylon, J., Fuller, J. N., van der Poel, C., Church, J. E., & Dworkin, S. (2022). Organophosphate insecticide toxicity in neural development, cognition, behaviour and degeneration: Insights from Zebrafish. *Journal of Developmental Biology,**10*(4), 49. 10.3390/jdb10040049 36412643 10.3390/jdb10040049 PMC9680476

[CR110] Ngole-Jeme, V. M., Ekosse, G.-I.E., & Songca, S. P. (2018). An analysis of human exposure to trace elements from deliberate soil ingestion and associated health risks. *Journal of Exposure Science & Environmental Epidemiology,**28*(1), 55–63. 10.1038/jes.2016.67 27924816 10.1038/jes.2016.67

[CR111] Nieder, R., Benbi, D. K., & Reichl, F. X. (2018). Soil as a transmitter of human pathogens. *Soil components and human health* (pp. 723–827). Dordrecht: Springer, Netherlands. 10.1007/978-94-024-1222-2_13

[CR112] Okore, N. K., Onyemeh, O. L., Remilekun, I. T., Melah, A. G., Gemadi, S. K., Elijah, E. U., & Ifebuzor, C. B. (2024). The effect of calabash chalk on the gastrointestinal motility and transit time of male albino Wistar rat. *World Journal of Advanced Research and Reviews,**24*(2), 425–434. 10.30574/wjarr.2024.24.2.3243

[CR113] Obuchowska, A., Gorczyca, K., Standyło, A., Obuchowska, K., Kimber-Trojnar, Ż, Wierzchowska-Opoka, M., & Leszczyńska-Gorzelak, B. (2022). Effects of probiotic supplementation during pregnancy on the future maternal risk of metabolic syndrome. *International Journal of Molecular Sciences,**23*(15), 8253. 10.3390/ijms23158253 35897822 10.3390/ijms23158253 PMC9330652

[CR114] Oh, C., Keats, E., & Bhutta, Z. (2020). Vitamin and mineral supplementation during pregnancy on maternal, birth, child health and development outcomes in low- and middle-income countries: A systematic review and meta-analysis. *Nutrients,**12*(2), 491. 10.3390/nu12020491 32075071 10.3390/nu12020491 PMC7071347

[CR115] Olajide-Kayode, J. O., Kolawole, T. O., Oyaniran, O. O., Mustapha, S. O., & Olatunji, A. S. (2023). Potentially harmful element toxicity in geophagic clays consumed in parts of southeastern Nigeria. *Journal of Trace Elements and Minerals,**4*, Article 100050. 10.1016/j.jtemin.2023.100050

[CR116] Olisa, O. G., Olajide-Kayode, J. O., Adebayo, B. O., Ajayi, O. A., Odukoya, K., Olalemi, A. A., & Uyakunmor, T.D.-M. (2023). Mineralogy and geochemical characterization of geophagic clays consumed in parts of southern Nigeria. *Journal of Trace Elements and Minerals,**4*, Article 100063. 10.1016/j.jtemin.2023.100063

[CR117] Orisakwe, O. E., Udowelle, N. A., Azuonwu, O., Nkeiruka, I. Z., Nkereuwem, U. A., & Frazzoli, C. (2020). Cadmium and lead in geophagic clay consumed in Southern Nigeria: Health risk from such traditional nutraceutical. *Environmental Geochemistry and Health,**42*(11), 3865–3875. 10.1007/s10653-020-00632-0 32607698 10.1007/s10653-020-00632-0

[CR118] Ortiz-Garcia, N. Y., Cipriano Ramírez, A. I., Juarez, K., Brand Galindo, J., Briceño, G., & Calderon Martinez, E. (2023). Maternal exposure to arsenic and its impact on maternal and fetal health: A review. *Cureus*. 10.7759/cureus.49177 10.7759/cureus.49177 PMC10734558 38130554

[CR119] Owhorji, B., Okon, U., Nwankwo, A., & Osim, E. (2019). Chronic consumption of calabash chalk diet impairs locomotor activities and social behaviour in Swiss white Cd-1 mice. *Heliyon,**5*(6), Article e01848. 10.1016/j.heliyon.2019.e01848 31194125 10.1016/j.heliyon.2019.e01848 PMC6551470

[CR120] Oyebanjo, O., Ekosse, G.-I., & Odiyo, J. (2020). Health Risk evaluation of trace elements in geophagic kaolinitic clays within Eastern Dahomey and Niger Delta Basins, Nigeria. *International Journal of Environmental Research and Public Health,**17*(13), 4813. 10.3390/ijerph17134813 32635450 10.3390/ijerph17134813 PMC7369916

[CR121] Parven, A., Meftaul, I. M., Venkateswarlu, K., & Megharaj, M. (2024). Herbicides in modern sustainable agriculture: Environmental fate, ecological implications, and human health concerns. *International Journal of Environmental Science and Technology*. 10.1007/s13762-024-05818-y

[CR122] Perković, S., Paul, C., Vasić, F., & Helming, K. (2022). Human health and soil health risks from heavy metals, micro(nano)plastics, and antibiotic resistant bacteria in agricultural soils. *Agronomy,**12*(12), 2945. 10.3390/agronomy12122945

[CR123] Prahl, M., Odorizzi, P., Gingrich, D., Muhindo, M., McIntyre, T., Budker, R., et al. (2021). Exposure to pesticides in utero impacts the fetal immune system and response to vaccination in infancy. *Nature Communications,**12*(1), 132. 10.1038/s41467-020-20475-8 10.1038/s41467-020-20475-8 PMC7794579 33420104

[CR124] Public Health England. (2013). Alert issued to pregnant women in London following reports of toxic ‘antidote’ to morning sickness. *Press release*. https://www.gov.uk/government/news/alert-issued-to-pregnant-women-in-london-following-reports-of-toxic-antidote-to-morning-sickness

[CR125] Puche-Juarez, M., Toledano, J. M., Moreno-Fernandez, J., Gálvez-Ontiveros, Y., Rivas, A., Diaz-Castro, J., & Ochoa, J. J. (2023). The role of endocrine disrupting chemicals in gestation and pregnancy outcomes. *Nutrients,**15*(21), 4657. 10.3390/nu15214657 37960310 10.3390/nu15214657 PMC10648368

[CR126] Rabapane, K. J., Ijoma, G. N., & Matambo, T. S. (2022). Insufficiency in functional genomics studies, data, and applications: A case study of bio-prospecting research in ruminant microbiome. *Frontiers in Genetics,**13*, Article 946449. 10.3389/fgene.2022.946449 36118848 10.3389/fgene.2022.946449 PMC9472250

[CR127] Richardson, J. R., Fitsanakis, V., Westerink, R. H. S., & Kanthasamy, A. G. (2019). Neurotoxicity of pesticides. *Acta Neuropathologica,**138*(3), 343–362. 10.1007/s00401-019-02033-9 31197504 10.1007/s00401-019-02033-9 PMC6826260

[CR128] Rosso, F., Rebellón-Sánchez, D. E., Llanos-Torres, J., Hurtado-Bermudez, L. J., Ayerbe, L., Suárez, J. H., et al. (2023). Clinical and microbiological characterization of Salmonella spp. isolates from patients treated in a university hospital in South America between 2012–2021: A cohort study. *BMC Infectious Diseases,**23*(1), 625. 10.1186/s12879-023-08589-y 37749501 10.1186/s12879-023-08589-y PMC10519077

[CR129] Saha, L., Kumar, V., Tiwari, J., Sweta, Rawat, & S., Singh, J., & Bauddh, K. (2021). Electronic waste and their leachates impact on human health and environment: Global ecological threat and management. *Environmental Technology & Innovation,**24*, Article 102049. 10.1016/j.eti.2021.102049

[CR130] Sarkar, T., Salauddin, M., Kirtonia, K., Pati, S., Rebezov, M., Khayrullin, M., et al. (2022). A review on the commonly used methods for analysis of physical properties of food materials. *Applied Sciences,**12*(4), 2004. 10.3390/app12042004

[CR131] Satarug, S. (2024). Is chronic kidney disease due to cadmium exposure inevitable and can it be reversed? *Biomedicines,**12*(4), 718. 10.3390/biomedicines12040718 38672074 10.3390/biomedicines12040718 PMC11048639

[CR132] Simonnet-Laprade, C., Bayen, S., Le Bizec, B., & Dervilly, G. (2021). Data analysis strategies for the characterization of chemical contaminant mixtures. Fish as a case study. *Environment International,**155*, Article 106610. 10.1016/j.envint.2021.106610 33965766 10.1016/j.envint.2021.106610

[CR133] Singh, A., Sharma, A., K. Verma, R., L. Chopade, R., P. Pandit, P., Nagar, V., et al. (2022). Heavy metal contamination of water and their toxic effect on living organisms. In *The Toxicity of Environmental Pollutants*. IntechOpen. 10.5772/intechopen.105075

[CR134] Stevens, E. J., Bates, K. A., & King, K. C. (2021). Host microbiota can facilitate pathogen infection. *PLOS Pathogens,**17*(5), Article e1009514. 10.1371/journal.ppat.1009514 33984069 10.1371/journal.ppat.1009514 PMC8118302

[CR135] Teffera, M., Veith, A. C., Ronnekleiv-Kelly, S., Bradfield, C. A., Nikodemova, M., Tussing-Humphreys, L., & Malecki, K. (2024). Diverse mechanisms by which chemical pollutant exposure alters gut microbiota metabolism and inflammation. *Environment International,**190*, Article 108805. 10.1016/j.envint.2024.108805 38901183 10.1016/j.envint.2024.108805 PMC12024183

[CR136] Tenebe, I. T., Babatunde, E. O., Eddy-Ugorji, C. C., Etu, E.-E.E., Ogarekpe, N. M., Ekeanyanwu, C. V., et al. (2023). Bacterial contamination levels and brand perception of sachet water: A case study in some Nigerian urban neighborhoods. *Water,**15*(9), 1762. 10.3390/w15091762

[CR137] ter Borg, S., Koopman, N., & Verkaik-Kloosterman, J. (2023). An evaluation of food and nutrient intake among pregnant women in The Netherlands: A systematic review. *Nutrients,**15*(13), 3071. 10.3390/nu15133071 37447397 10.3390/nu15133071 PMC10346763

[CR138] Ventura-Miranda, M. I., Fernández-Medina, I. M., Guillén-Romera, E., Ortíz-Amo, R., & Ruíz-Fernández, M. D. (2022). Effect of gestational pesticide exposure on the child’s respiratory system: A narrative review. *International Journal of Environmental Research and Public Health,**19*(22), 15418. 10.3390/ijerph192215418 36430137 10.3390/ijerph192215418 PMC9690583

[CR139] Vofo, B. N., Fotsing Ngankam Vofo, G. V., Ambo Fonge, B., Nsagha, D. S., Obinchemti Egbe, T., & Nguedia, J. C. (2019). High umbilical cord blood lead levels and “calabar chalk” consumption amongst pregnant women in two hospitals in Cameroon. *Pan African Medical Journal*, *33*. 10.11604/pamj.2019.33.109.13999 10.11604/pamj.2019.33.109.13999 PMC6711677 31489087

[CR140] Wallace, D. R., & Buha Djordjevic, A. (2020). Heavy metal and pesticide exposure: A mixture of potential toxicity and carcinogenicity. *Current Opinion in Toxicology,**19*, 72–79. 10.1016/j.cotox.2020.01.001

[CR141] Webale, M. K., Wanjala, C., Guyah, B., Shaviya, N., Munyekenye, G. O., Nyanga, P. L., et al. (2020). Epidemiological patterns and antimicrobial resistance of bacterial diarrhea among children in Nairobi City, Kenya. *Gastroenterology and Hepatology from Bed to Bench,**13*(3), 238–246. 10.22037/ghfbb.v13i3.1910 32821354 PMC7417493

[CR142] Wei, S. Q., Bilodeau-Bertrand, M., Liu, S., & Auger, N. (2021). The impact of COVID-19 on pregnancy outcomes: A systematic review and meta-analysis. *Canadian Medical Association Journal,**193*(16), E540–E548. 10.1503/cmaj.202604 33741725 10.1503/cmaj.202604 PMC8084555

[CR143] Wongstitwilairoong, B., Anothaisintawee, T., Ruamsap, N., Lertsethtakarn, P., Kietsiri, P., Oransathid, W., et al. (2023). Prevalence of intestinal parasitic infections, genotypes, and drug susceptibility of Giardia Lamblia among preschool and school-aged children: A cross-sectional study in Thailand. *Tropical Medicine and Infectious Disease,**8*(8), 394. 10.3390/tropicalmed8080394 37624332 10.3390/tropicalmed8080394 PMC10457730

[CR144] Wyckhuys, K. A. G., Aebi, A., Bijleveld van Lexmond, M. F. I. J., Bojaca, C. R., Bonmatin, J.-M., Furlan, L., et al. (2020). Resolving the twin human and environmental health hazards of a plant-based diet. *Environment International,**144*, Article 106081. 10.1016/j.envint.2020.106081 32889485 10.1016/j.envint.2020.106081

[CR145] Xiao, W., Zhang, J., Huang, J., Xin, C., Li, M. J., & Song, Z. (2022). Response and regulatory mechanisms of heat resistance in pathogenic fungi. *Applied Microbiology and Biotechnology,**106*(17), 5415–5431. 10.1007/s00253-022-12119-2 35941254 10.1007/s00253-022-12119-2 PMC9360699

[CR146] Yemmireddy, V., Adhikari, A., & Moreira, J. (2022). Effect of ultraviolet light treatment on microbiological safety and quality of fresh produce: An overview. *Frontiers in Nutrition,**9*, Article 871243. 10.3389/fnut.2022.871243 35942168 10.3389/fnut.2022.871243 PMC9356256

[CR147] Zhang, Y.-J., Li, S., Gan, R.-Y., Zhou, T., Xu, D.-P., & Li, H.-B. (2015). Impacts of gut bacteria on human health and diseases. *International Journal of Molecular Sciences,**16*(4), 7493–7519. 10.3390/ijms16047493 25849657 10.3390/ijms16047493 PMC4425030

[CR148] Zhong, Z., Yang, Q., Li, C., Chen, X., & Zhou, F. (2022). A global perspective of correlation between maternal blood lead levels and risks of preeclampsia: An updated systematic review and meta-analysis. *Frontiers in Public Health,**10*, Article 1072052. 10.3389/fpubh.2022.1072052 36620238 10.3389/fpubh.2022.1072052 PMC9816335

